# Biocompatibility of Hydrogels for Glomerular 3D Co‐Culture: A Comparative Analysis

**DOI:** 10.1002/mabi.202500460

**Published:** 2026-02-24

**Authors:** Julia Eichermüller, Jessica Faber, Xuen Ng, Camilla Mussoni, Julian Bauer, Jonas Röder, Alessandro Cianciosi, Philipp Stahlhut, Tomasz Jungst, Jürgen Groll, Dominik Steiner, Thomas Scheibel, Oliver Friedrich, Taufiq Ahmad, Aldo R. Boccaccini, Silvia Budday, Janina Müller‐Deile

**Affiliations:** ^1^ Department of Nephrology Uniklinikum Erlangen Friedrich‐Alexander‐Universität Erlangen‐Nürnberg Erlangen Germany; ^2^ Institute of Continuum Mechanics and Biomechanics Friedrich‐Alexander‐Universität Erlangen‐Nürnberg Fürth Germany; ^3^ Department of Biomaterials University of Bayreuth Bayreuth Germany; ^4^ Department of Functional Materials in Medicine and Dentistry Institute of Biofabrication and Functional Materials University of Würzburg Würzburg Germany; ^5^ Institute of Medical Biotechnology Department of Chemical and Biological Engineering Friedrich‐Alexander‐Universität Erlangen‐Nürnberg Erlangen Germany; ^6^ Department of Materials Science Friedrich‐Alexander‐Universität Erlangen‐Nürnberg Erlangen Germany; ^7^ Department of Plastic and Hand Surgery BG Clinic Tübingen University of Tübingen Tübingen Germany

**Keywords:** ADA‐GEL, fibrin gel, GelAGE, glomerular co‐culture, matrigel, spider silk

## Abstract

Conventional 2D mono‐cultures fall short in replicating the complex microenvironment of glomerular tissue, where cell–cell and cell–matrix interactions are critical. To better mimic *in vivo* conditions, the development of robust 3D co‐culture systems is essential. Here, we systematically evaluate five hydrogel matrices—Matrigel, alginate dialdehyde‐gelatin (ADA‐GEL), fibrin, recombinantly produced spider silk protein eADF4(C16)‐RGD, and allyl‐modified gelatin (GelAGE)—for their suitability in supporting glomerular 3D co‐culture. The hydrogels are assessed for handling properties, cell viability, and the support of physiological cell behavior using bright‐field microscopy, live/dead assays, immunofluorescence, and multiphoton imaging. Among the tested hydrogels, GelAGE and eADF4(C16)‐RGD demonstrate superior biocompatibility and structural support. Due to its ease of use and comparable biological performance, GelAGE and spider silk protein eADF(C16)‐RGD are selected for further mechanical characterization, revealing favorable viscoelastic properties. These findings position both hydrogels as a promising candidate for engineering physiologically relevant 3D glomerular models and advancing kidney tissue research.

## Graphical abstract

GelAGE and recombinant spider silk hydrogels are evaluated for glomerular 3D co‐culture. GelAGE combines ease of handling with excellent biocompatibility and viscoelasticity, making it a promising scaffold for kidney tissue modeling and advanced *in vitro* studies.

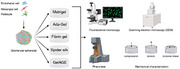

## 1 Introduction

The kidney's functional unit is the nephron, which consists of one glomerulus, where primary urine is filtered from the blood, and a tubular system where vital substances like water, ions, and amino acids are reabsorbed back into the bloodstream, and waste products are concentrated into urine. The glomerular filtration barrier of the kidney comprises glomerular endothelial cells, the glomerular basement membrane, and podocytes with interdigitating foot processes and a slit membrane between them [1, 2]. Furthermore, mesangial cells within the glomerulus are in cell‐cell communion with glomerular endothelial cells and podocytes and maintain the glomerular structure by producing an extracellular matrix (ECM) [3].

For a better understanding of glomerular signal transduction, various cell culture models were generated in the past, mostly 2D models [4]. However, 2D cell cultures cannot adequately recapitulate the complex glomerular structure. 3D self‐assembling glomerular spheroids have been established in our lab before and showed an enhanced expression of genes important for glomerular function in 3D compared to 2D [5]. However, so far, these spheroids lack vascularization and environmental cues, thus limiting their application for higher hierarchical glomerular‐like structures.

A promising strategy to solve this problem is the generation of bioartificial tissues by seeding cells in hydrogels. Hydrogels are 3D polymer networks with a water content of above 95% (w/w) and high swelling rates [6, 7]. They allow for cell seeding in a highly hydrated, mechanically supportive 3D environment. Hydrogels have proven useful in a wide range of cell culture applications, including tissue engineering [8, 9]. However, the hydrogel of choice must fulfill certain material characteristics, which depend on different cellular needs and functions of the tissue to be simulated.

As mechanical, structural, and compositional properties differ between hydrogel types and since they can markedly alter cell function, it is important to test and analyze them with the cell types of interest before using them in later models. Hence, a wise selection of hydrogels is important. This includes biocompatibility, no toxicity, no immunogenicity, biodegradability, and suitable pore sizes enabling cells to receive nutrition and oxygen and allowing cell‐cell communication.

Considering the type of tissue to be engineered, the mechanical properties of the scaffold also have to be taken into account. Even though multiple hydrogels have been tested in different cell culture models, especially for tumor and tissue regeneration, a systematic comparison of different hydrogels in 3D co‐culture of different glomerular cell types has never been performed before [10, 11]. It is unknown which hydrogel fits best for glomerular 3D co‐culture experiments.

In this project, we simulated the 3D context of glomerular cells using spheroids composed of immortalized human podocytes, glomerular endothelial cells, and mesangial cells embedded in different hydrogels. This study aimed to identify the best hydrogel, providing an ECM‐mimicking environment to ensure high cell survival of glomerular co‐cultures over time to allow vascularization in the future. For this purpose, Matrigel, alginate dialdehyde‐gelatin (ADA‐GEL), fibrin gel, spider silk, and allyl‐modified gelatin (GelAGE) were tested. Initially, glomerular cells were tested in mono‐cultures, but only when all three cell types of the co‐culture survived in the hydrogel, and, if gel stability allowed cultivation for longer periods of time, only then was the gel considered for further experiments. Multimodal mechanical measurements determined the complex mechanical properties of cell‐laden GelAGE and spider silk hydrogels over 14 days, and cryo‐SEM imaging provided insights into the pore size and structure of the matrices. Live/dead and cytoskeleton staining provided information on viability, cell morphology, cell spreading, and interactions.

## 2 Results

To provide a morphological reference, representative images of 2D cultures on tissue culture plastic were included for all cell types. These serve as a direct comparison to the corresponding 3D hydrogel cultures, highlighting the distinct differences in cell morphology and organization between 2D and 3D environments (Figure S1). Each hydrogel was initially seeded with human mesangial cells in mono‐culture to assess the basic performance and biocompatibility of the material, as mesangial cells are the least demanding of the three glomerular cell types and thus serve as a suitable first indicator of gel compatibility.

### 2.1 Matrigel as an ECM‐Mimicking Environment for Glomerular Cells

**FIGURE 1:**
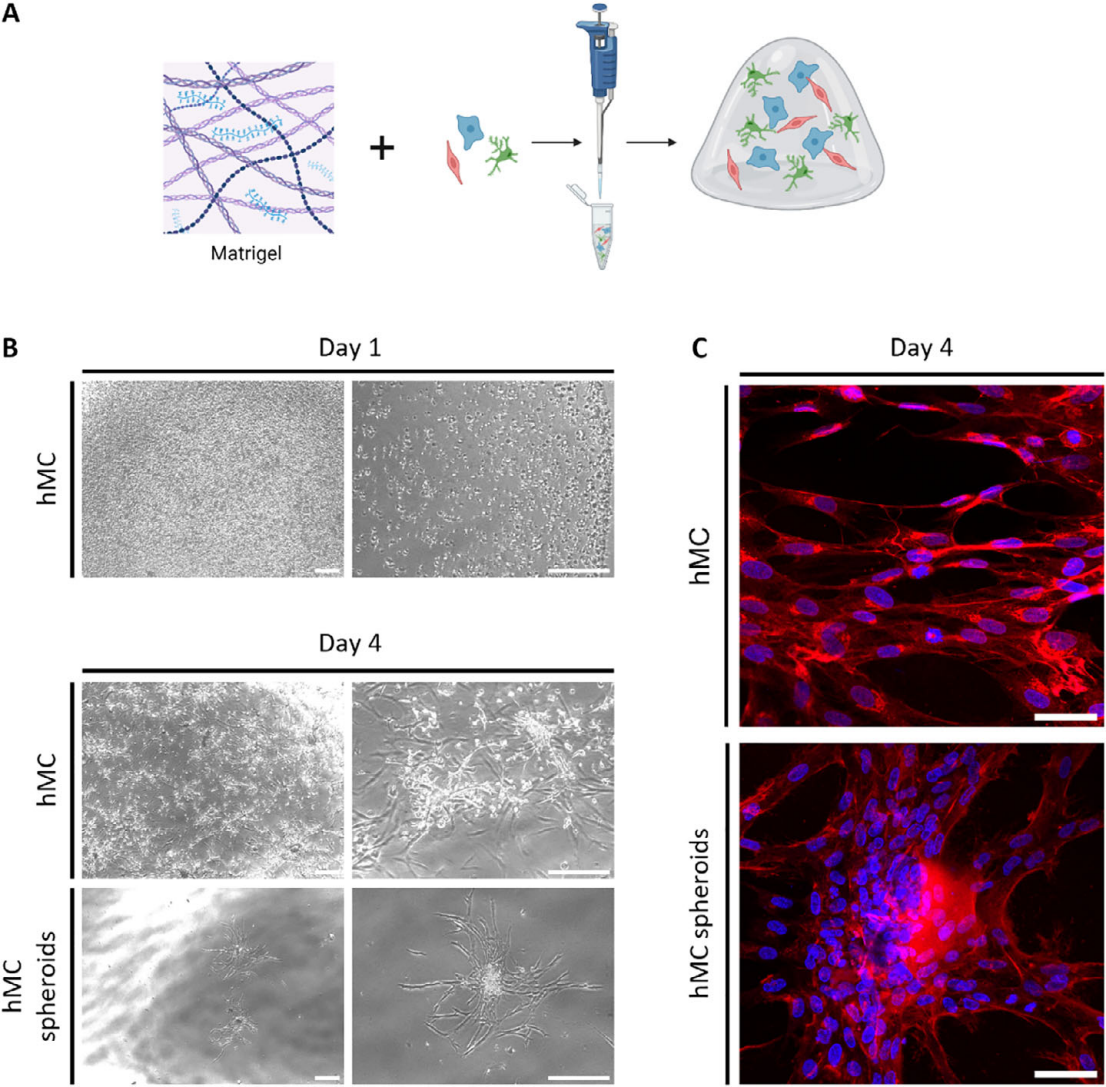
Matrigel as an ECM‐mimicking environment for glomerular cells. (A) Schematic illustration of Matrigel hydrogel fabrication with encapsulated glomerular cells. (B) Bright‐field microscopy of hMC in Matrigel domes on coverslips after one (upper panel) and four days (middle and lower panels). The middle panel shows the formation of the characteristic growth pattern of hMCs. The lower panel reveals self‐formation of 3D mono‐culture spheroids. Scale bars: left side: 750 µm and right side: 300 µm. (C) Confocal microscopy of 2D hMC (top) and 3D hMC spheroids embedded in Matrigel domes (bottom) after four days depicts cell elongation and cell‐cell contacts. Nuclei were stained with Hoechst 33342 (blue), and cell surface marker WGA (red). Scale bar: 50 µm. ECM: extracellular matrix, hMC: human mesangial cells, WGA: wheat germ agglutinin. Experiments with Matrigel were performed more than three times.

Matrigel, a commercially available solubilized basement membrane matrix derived from mouse sarcoma cells, was evaluated as the first hydrogel. Rich in laminin and collagen IV, Matrigel represents a promising scaffold for glomerular cell culture (Figure 1A) [12]. Mesangial cells encapsulated in Matrigel remained viable, proliferated, and displayed spreading behavior. After four days of culture, the cells self‐organized into 3D spheroid‐like structures. When pre‐formed human mesangial cell spheroids were encapsulated, cell spreading and outgrowth from the spheroids were detectable within four days (Figure 1B, lower panel; Figure S2). Wheat germ agglutinin (WGA) staining and confocal microscopy further revealed cell elongation and the formation of cell‐cell contacts within Matrigel (Figure 1C).

However, due to rapid gel degradation that prevented medium exchange, fixation, and staining, Matrigel was deemed unsuitable for continued 3D glomerular co‐culture experiments, and no further studies were conducted with this hydrogel.

### 2.2 ADA‐GEL as an ECM‐Mimicking Environment for Glomerular Cells

**FIGURE 2:**
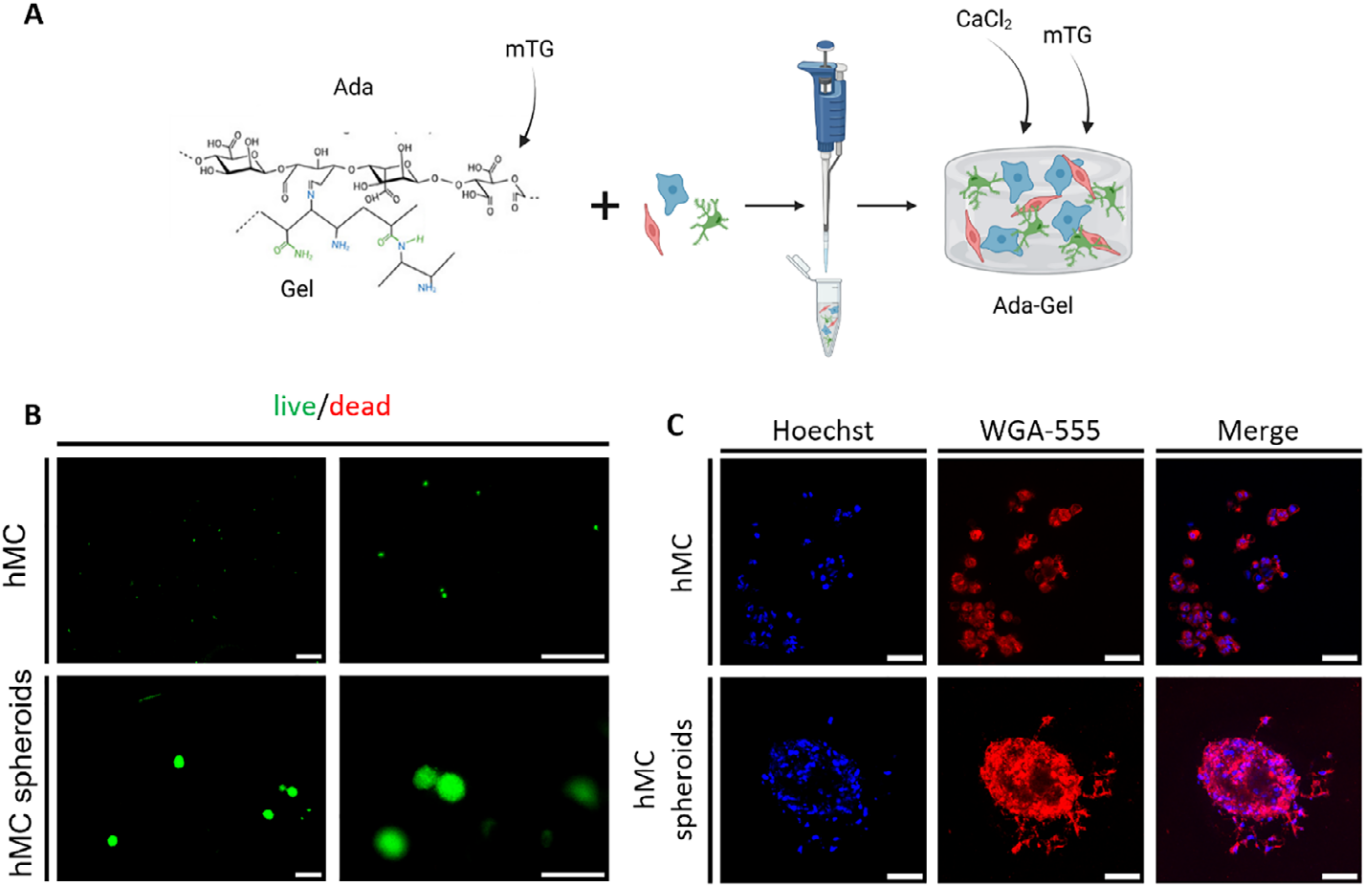
ADA‐GEL as an ECM‐mimicking environment for glomerular cells. (A) Schematic illustration of ADA‐GEL hydrogel fabrication with encapsulated glomerular cells. (B) Viability of hMCs grown as 2D culture (upper panel) and 3D mono‐culture (lower panel) in ADA‐GEL using live (green)/dead (red) fluorescence staining after five days imaged by fluorescence microscopy. Scale bars: 300 µm. (C) Morphology of hMCs using confocal microscopy (upper panel: 2D cultivation, lower panel: 3D mono‐culture, blue: Hoechst 33342, red: WGA, right: merge). Scale bar: 50 µm. ADA‐GEL: alginate dialdehyde‐gelatin, ECM: extracellular matrix, hMC: human mesangial cells, WGA: wheat germ agglutinin. Experiments with ADA‐GEL were performed more than three times.

Gelatin alone lacks sufficient thermal stability both *in vitro* and *in vivo*. To address this limitation, we combined gelatin with alginate dialdehyde (ADA), which allows for ionic crosslinking via calcium ions, thereby enhancing the structural stability of the hydrogel. As a second matrix, oxidized alginate‐gelatin (ADA‐GEL) was initially used for glomerular mono‐cultures. ADA‐GEL by itself rapidly degraded after four days of cultivation with encapsulated hMC cells (data not shown). The addition of microbial transglutaminase (mTG) provided a secondary crosslinking mechanism, specifically targeting the gelatin component rather than the ADA (Figure 2A). This enzymatic crosslinking further improves the mechanical integrity of the composite hydrogel [13].

However, variances between the batches of ADA‐GEL were too high for reproducible consistency of the hydrogel. Hence, gels were unstable, but when mTG and CaCl_2_ were added, the degradation of the matrix was slowed down extensively (Figure S5), but cells were affected in their proliferation behavior.

ADA‐GEL hydrogels were assessed for their suitability for glomerular cell culture. Gels were too opaque to perform clear bright field images of cells within the gel (Figure S3). Live/dead staining of mesangial cells revealed viable cells after five days; however, only a subset remained within the hydrogel, as the majority spread out of the matrix (Figure 2B, upper panels), whereas pre‐formed mesangial spheroids largely remained in place (Figure 2B, lower panels). Confocal microscopy showed minimal sprouting from both single‐cell suspensions and spheroids, indicating active cell spreading within the hydrogel. Wheat germ agglutinin (WGA) staining revealed that mesangial cells in 2D culture remained mostly rounded with limited proliferation (Figure 2C, upper panel, Figure S4), whereas 3D spheroid cultures exhibited cellular extensions and sprouting (Figure 2C, lower panel).

In summary, ADA‐GEL proved challenging for further experiments: gels continuously dissolved during culture without the addition of CaCl_2_ and mTG, but when used, the cells were unable to proliferate and migrate at the desired rate to sufficiently form tissue‐like structures, limiting studies with glomerular cells or co‐cultures. ADA‐GELs showed significant batch‐to‐batch variability, undermining reproducibility.

In conclusion, ADA‐GEL is unsuitable for glomerular 3D co‐culture due to the inconsistency of stability, insufficient transparency, and cytotoxic effects on glomerular endothelial cells. Future work could explore alternative ADA‐GEL formulations, including variations in oxidation degree, ADA‐to‐GEL ratios, or modified cross‐linking strategies (e.g., pre‐cross‐linking), as described elsewhere, to improve hydrogel stability and cell compatibility [14].

### 2.3 Fibrin Gel as an ECM‐Mimicking Environment for Glomerular Cells

**FIGURE 3:**
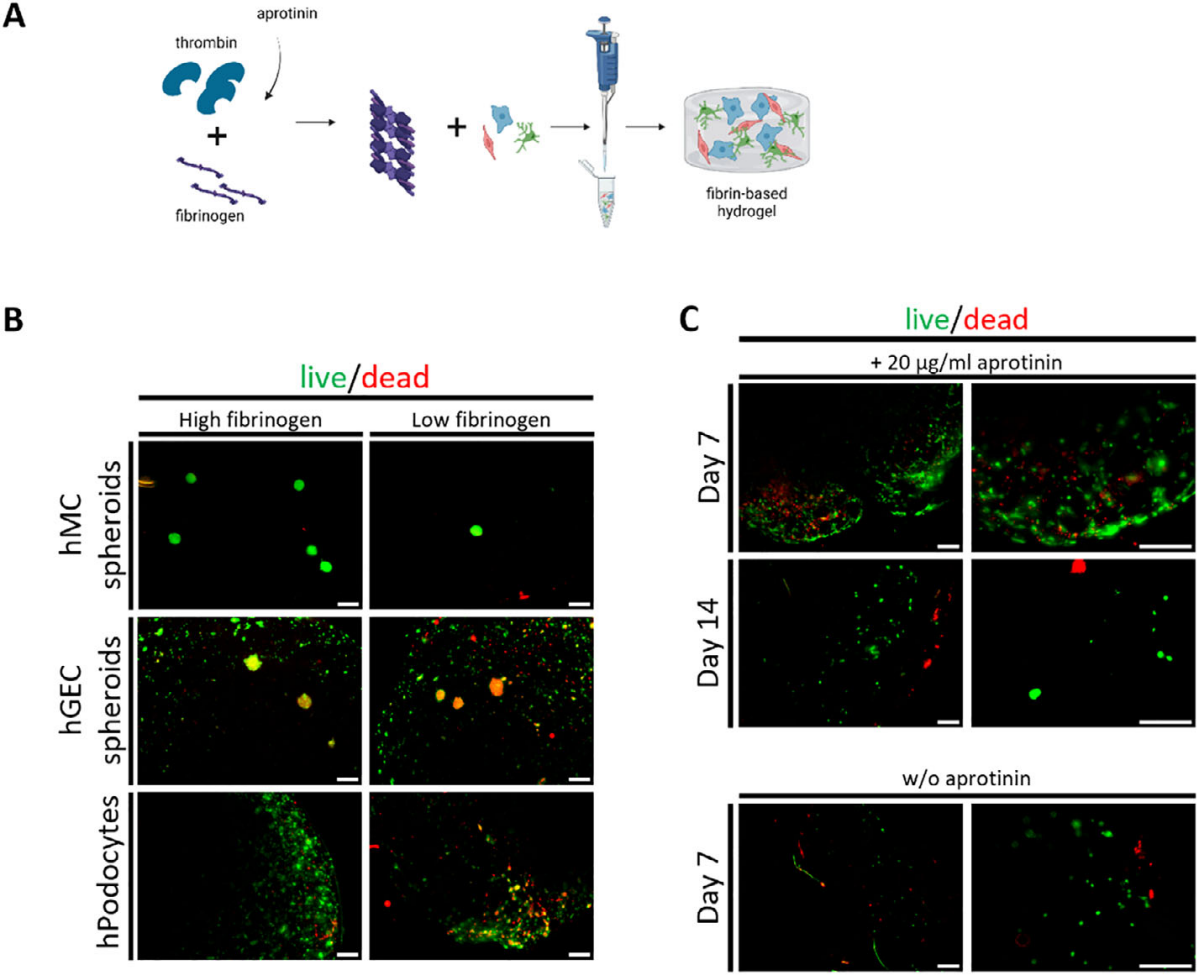
Fibrin gel as an ECM‐mimicking environment for glomerular cells. (A) Schematic illustration of fibrin gel fabrication from fibrinogen and thrombin with encapsulated glomerular cells. (B) Viability of hMC spheroids, hGEC spheroids, and hPodocytes in fibrin gels composed of high fibrinogen (left panel) and low fibrinogen (right panel) using live (green)/dead (red) fluorescence staining after one day of culture imaged by fluorescence microscopy. Scale bar: 300 µm. (C) Viability of podocytes in fibrin gels composed of high fibrinogen with (upper panel) and without (lower panel) aprotinin using live (green)/dead (red) fluorescence staining after seven and 14 days of culture imaged by fluorescence microscopy. Scale bar: left side: 750 µm and right side: 300 µm. ECM: extracellular matrix, hMC: human mesangial cells, hGEC: human glomerular endothelial cells, hPodocytes: immortalized human podocytes. Experiments with fibrin were performed more than three times.

Next, fibrin was tested in concentrations that have been previously published [15], as a hydrogel for glomerular culture. Fibrin has been used in a variety of biomaterials, cell delivery, and tissue engineering applications [16, 17]. Fibrin gels were generated by adding thrombin in a concentration of 50 IU/mL to fibrinogen. Thrombin catalyzes the formation of fibrin microfibrils, which form a 3D mesh (Figure 3A) [18]. A low (15 mg/mL) and a high concentration (22.75 mg/mL) of fibrinogen were mixed with 50 IU/mL thrombin to form fibrin. Similar to ADA‐GEL, fibrin‐based gels were opaque, hindering clear bright‐field imaging, and exhibited gradual degradation over time (Figure S6). Fluorescence based live/dead staining revealed that human mesangial cells and human glomerular endothelial cells survived in fibrin gels with higher fibrinogen content, and 3D structures of the cells were able to spread within the gel. Human podocytes also survived in the fibrin gel, even though these cells could not be grown into spheroids since this cell type does not form stable mono‐culture spheroids (Figure 3B). Therefore, the higher concentration of fibrinogen was used for further experiments with fibrin‐based hydrogels. However, the fibrin gel quickly degraded (within seven days), rendering cell culture over several days impossible. Next, the protease inhibitor aprotinin was added to the gels. Despite increased fibrin gel stability until day 14 (compared to only a maximum of seven days without aprotinin), good cell morphology and cell survival of podocytes markedly decreased, making this approach useless for glomerular tissue engineering (Figure 3C). Another limitation of the fibrin gel was its poor optical transparency, which impeded clear immunofluorescence imaging.

Therefore, fibrin was not considered suitable for supporting glomerular 3D co‐culture systems.

### 2.4 Spider Silk as an ECM‐Mimicking Environment for Glomerular Co‐Culture

**FIGURE 4:**
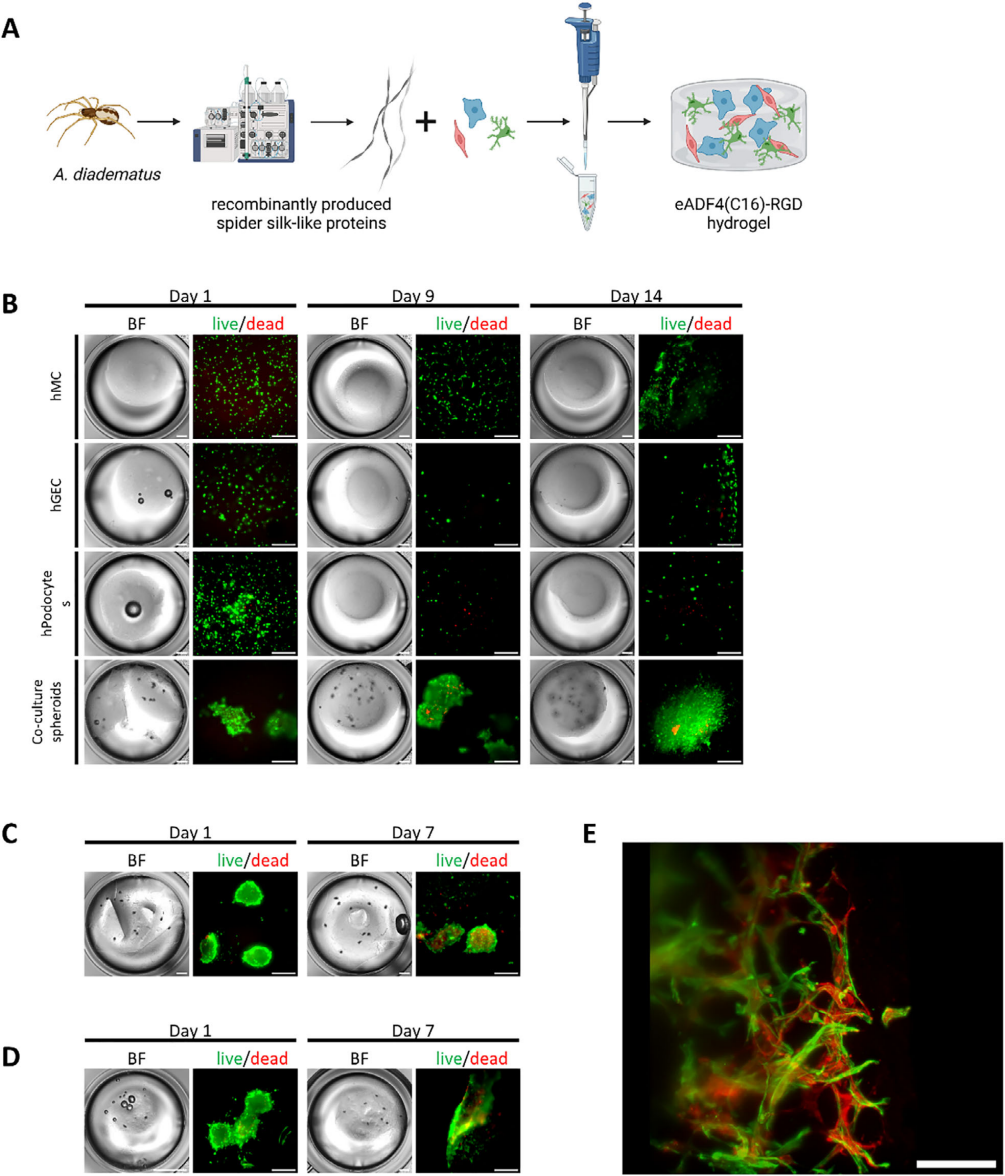
Spider silk as an ECM‐mimicking environment for glomerular co‐culture. (A) Schematic illustration of recombinantly produced spider silk‐like proteins for hydrogel fabrication with encapsulated glomerular cells. (B) Bright‐field (left panels) and live (green)/dead (red) fluorescence staining (right panels) of hMC, hGEC, and hPodocytes and 3D glomerular co‐culture of hMC, hGEC, and hPodocytes in spider silk‐based hydrogels. Imaged after one day, nine days, and 14 days of culture. Scale bars: Bright‐field: 1,000 µm and live/dead: 300 µm. (C) Bright‐field (left panels) and live (green)/dead (red) fluorescence staining (right panels) of 3D glomerular co‐culture of hMC, hGEC, and hPodocytes with incorporated VEGF‐A coated fibers in spider silk‐based hydrogels. Imaged after one day and seven days of culture. Scale bars: Bright‐field: 1,000 µm and live/dead: 300 µm. (D) Bright‐field (left panels) and live (green)/dead (red) fluorescence staining (right panels) of 3D glomerular co‐culture of hMC, hGEC, and hPodocytes in spider silk collagen IV composite hydrogels. Imaged after one day and seven days of culture. Scale bars: Bright‐field: 1,000 µm and live/dead: 300 µm. (E) Light‐sheet microscopy image (after 14 days) of 3D glomerular co‐culture in spider silk stained with WGA (red) and Phalloidin (green). Scale bar: 500 µm. ECM: extracellular matrix, hMC: human mesangial cells, hGEC: human glomerular endothelial cells, hPodocytes: immortalized human podocytes, VEGF‐A: vascular endothelial growth factor‐A, WGA: wheat germ agglutinin. Experiments with spider silk were performed more than three times.

As spider silk is known for good biocompatibility, a spider silk‐based hydrogel was a promising candidate for a hydrogel for glomerular co‐culture [19]. Spider silk gel (3%) modified with RGD sequence was used to test human podocytes, human mesangial cells, and human glomerular endothelial cell mono‐cultures as well as co‐cultures (Figure 4A). Despite the relatively soft gel consistency [19], the spider silk‐based gel remained stable for more than three weeks, could be fixed with 4% paraformaldehyde, and stained. While 2D glomerular mono‐cultures exhibited only moderate survival in spider silk gels, 3D glomerular co‐cultures showed markedly superior viability compared to the other hydrogels (Figure 3B). By day 14, cells had migrated from the spheroids and formed an expanded, scaffold‐like structure (Figure 4E).

Next, we tested spider silk gels added with vascular endothelial growth factor‐A (VEGF‐A) coated fibers or composite gels with collagen IV to improve the glomerular ECM‐mimicking environment.

VEGF plays a pivotal role in promoting endothelial cell survival, proliferation, and angiogenic activity. Since hGECs do not produce VEGF‐A themselves, we hypothesized that a localized presentation of VEGF could enhance cell viability and functionality within the hydrogel. The inclusion of VEGF‐functionalized fibers aimed to mimic the native extracellular matrix and to provide spatially controlled, sustained pro‐survival signals directly within the hydrogel. The efficient coating of fibers with VEGF‐A and the kinetics of its release were described before [20]. In our study, over 99% of the VEGF was immobilized on the fibers, confirming efficient functionalization (Figure S7).

Co‐culture spheroids containing VEGF‐A‐coated poly‐L‐lactic acid (PLLA) fibers did not show the desired effect of improved cell viability (Figure 4C). Given the critical role of collagen IV as a key component of the glomerular basement membrane and glomerular extracellular matrix, composite hydrogels incorporating spider silk and collagen IV were tested to support the viability of glomerular spheroids. Likewise, no improvement in viability was observed in spheroids embedded in spider silk–collagen IV composite hydrogels (Figure 4D).

Glomerular co‐cultures of human mesangial cells, human glomerular endothelial cells, and podocytes were imaged with light‐sheet microscopy after 14 days of culture to obtain high‐resolution 3D images. Cells were stained with WGA to visualize the cell membrane and with phalloidin for actin cytoskeleton labeling. Cells grew out of the spheroids and formed a 3D scaffold‐like structure (Figure 4E; Video S1).

### 2.5 Mechanical Characteristics of Spider Silk Hydrogel with Encapsulated Glomerular Co‐Culture Spheroids

After establishing spider silk as a suitable hydrogel for glomerular co‐culture, we aimed to investigate how glomerular cells interact with the matrix and whether the gel's properties change over time. To this end, we assessed the influence of glomerular cells on the complex mechanical behavior of spider silk gels. Initial cyclic loading tests were performed on acellular gels to characterize baseline mechanical properties (Figure S8).

**FIGURE 5:**
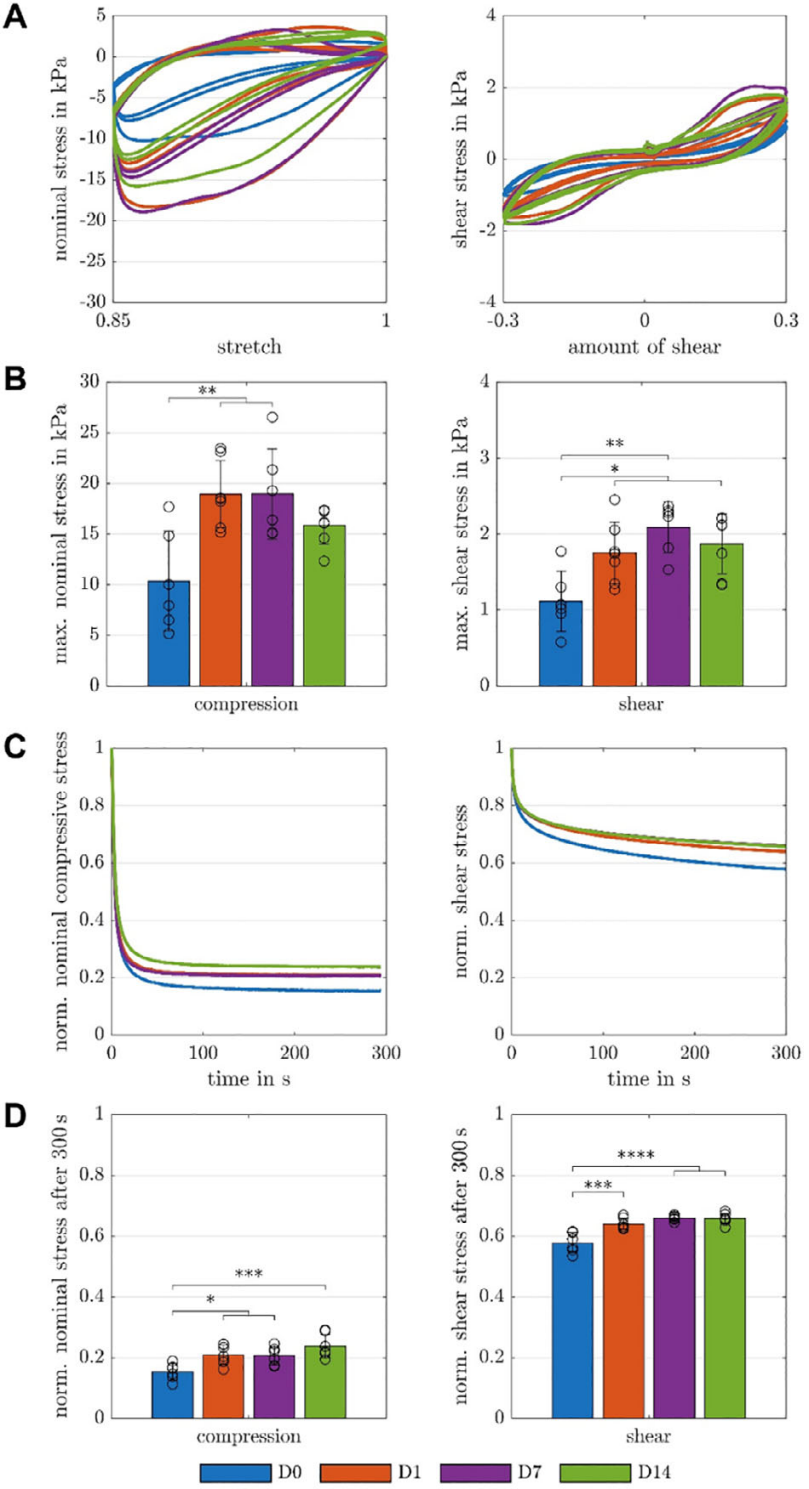
Mechanical characterization of spider silk hydrogel with encapsulated glomerular co‐culture. (A) Testing setup: Rheometer to perform multi‐modal mechanical testing of glued cylindrical spider silk samples in compression and torsional shear under large strains. (B) Cyclic loading behavior of cell‐laden spider silk hydrogels (D0, D7: *n* = 6, D1, D14: *n* = 7) during cyclic compression up to a maximum strain of 15% in compression (left), and during cyclic shear up to a maximum shear of 30% in torsional shear (right) over 14 days of cultivation. (C) Corresponding average maximum nominal stresses in compression (left), and torsional shear (right) over 14 days of cultivation. (D) Average normalized stress relaxation behavior of spider silk hydrogels with encapsulated glomerular co‐culture consisting of hMC, hGEC and hPodocytes at a maximum strain of 15% in compression (left), and at a maximum shear of 30% in torsional shear (right) over 14 days of cultivation. (E) Corresponding average normalized stress after 300 s relaxation in compression (left), and torsional shear (right) over 14 days of cultivation. Significances were calculated using one‐way ANOVA tests if all samples were normally distributed and Kruskal‐Wallis tests otherwise, followed by Tukey‐Kramer tests for multiple comparisons. Significance values: ^*^ *p* < 0.05, ^**^ *p* < 0.01, ^***^ *p* < 0.001, **** *p <* 0.0001. hMC: human mesangial cells, hGEC: human glomerular endothelial cells, hPodocytes: immortalized human podocytes.

To determine the influence of glomerular cells on the complex mechanical properties of spider silk hydrogels, cyclic loading and stress relaxation tests in compression, and torsional shear of spider silk hydrogels with encapsulated glomerular spheroids were performed (Figure 5A), and the complex mechanical characteristics, including nonlinearity, hysteresis, conditioning, and stress relaxation, were evaluated. Up to day 7, glomerular cell‐containing spider silk hydrogels showed a more pronounced nonlinear, hysteretic behavior with increasing nominal stresses during cyclic loading, but similar (pre)conditioning effects compared to day 0. The hysteretic behavior was evaluated by the hysteresis area, defined as the enclosed area between the loading and unloading curve of the stress‐stretch or stress‐shear response during each cycle, which quantifies the corresponding energy dissipation of each cycle. As the cell network matured over time, the nominal stresses significantly increased in compression, and torsional shear from day 0 to day 1/day 7 (Figure 5A, B). Only a slight, but not significant decrease in the nominal stresses in the mechanical properties from day 7 to 14 is visible.

During stress relaxation tests, glomerular cell‐containing spider silk hydrogels exhibited characteristic viscoelastic behavior and relaxed fast up to 85% in compression, and up to 42% in torsional shear on day 0, but did not reach equilibrium stress values in torsional shear after 300 s. A visible trend of continuous decrease in the viscous effects from day 0 to day 14 for all loading modes (Figure 5C, D) could be attributed to the maturation of cellular networks within the polymer chain network.

To evaluate changes in the spider silk hydrogels’ properties over time, we performed cyclic loading tests without cells and determined the complex mechanical properties, including nonlinearity, hysteresis, and conditioning (Figure S8). While a decrease in the nominal stresses, nonlinearity, hysteresis, and conditioning from day 0 to day 1 was visible in compression, the material properties remained similar up to day 14. In torsional shear, a slight, but not significant, continuous decrease in nominal stresses, nonlinearity, hysteresis, and conditioning is visible.

In summary, spider silk seems to be a hydrogel that is suitable for glomerular co‐culture experiments. However, spider silk material requires a highly qualified expert who can assess the two most important parameters, namely time and temperature, to ensure that the hydrogels provide the same quality, good handling properties, and durability of the samples. Without special readjustment, this hydrogel is more complex to use, and an adaptation to the respective laboratory conditions is required.

### 2.6 GelAGE as an ECM‐Mimicking Environment for Glomerular Co‐Culture

GelAGE offers excellent handling characteristics, being both optically transparent for high‐quality imaging and highly stable by UV‐crosslinked polymerization [21]. GelAGE‐based gel was generated by mixing the GelAGE variant G_2_LH [22], 4‐arm thiolated polyethylene glycol (PEG‐4‐SH), and lithium phenyl‐2,4,6‐trimethylbenzoylphosphinate (LAP) as the photoinitiator. The degree of modification was determined by ^1^H‐NMR spectroscopy (Figure S9).

**FIGURE 6:**
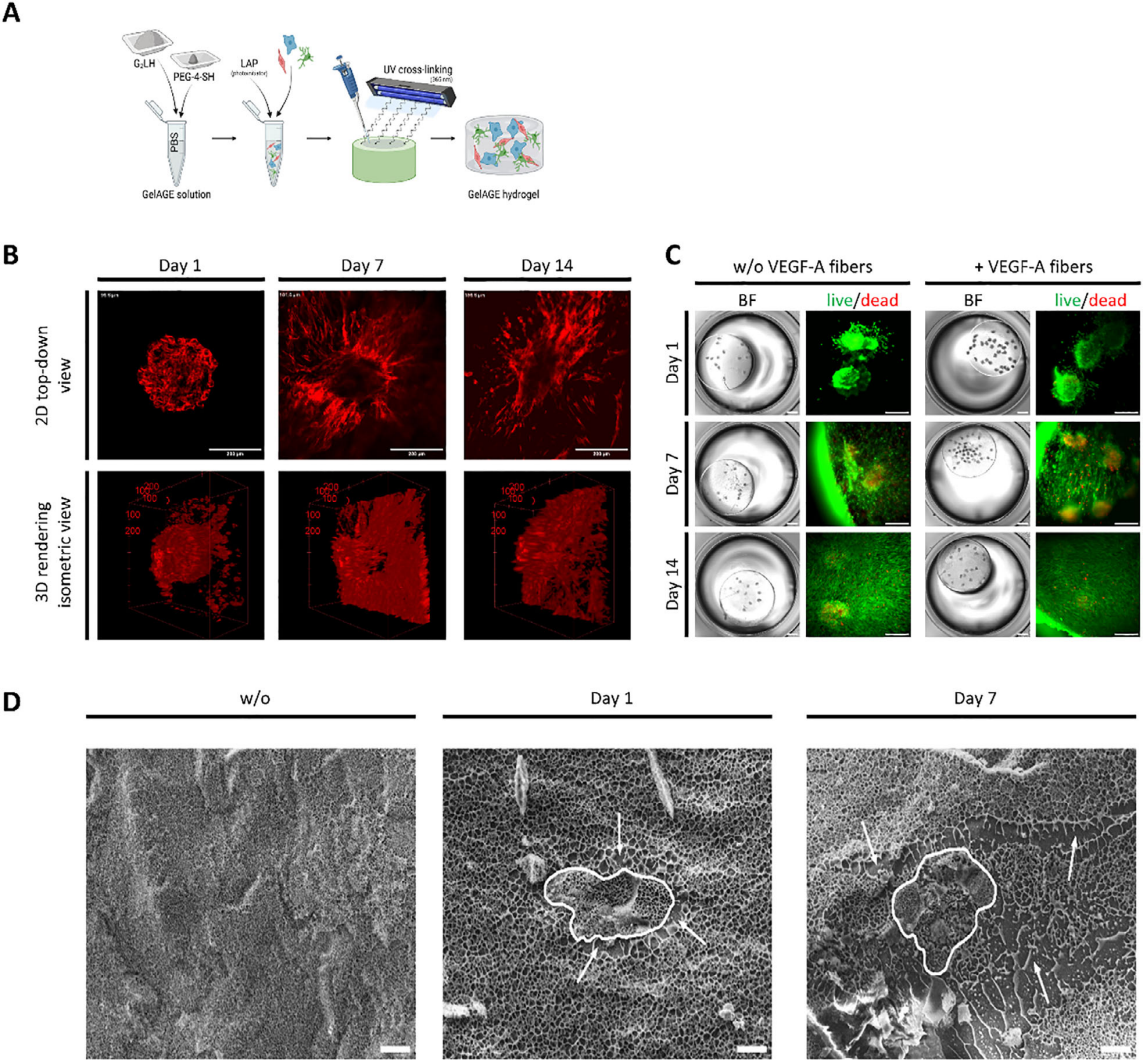
GelAGE as an ECM‐mimicking environment for glomerular co‐culture. (A) Schematic illustration of GelAGE hydrogel fabrication with encapsulated glomerular cells. (B) Multiphoton microscopy images of 3D glomerular co‐culture in GelAGE labeled with phalloidin after one day, seven days, and 14 days of culture. Scale bar: 200 µm. (C) Bright‐field (left panels) and live (green)/dead (red) fluorescence staining (right panels) of 3D glomerular co‐culture of hMC, hGEC, and hPodocytes in GelAGE‐based hydrogels without VEGF‐A coated fibers (left panel) and with VEGF‐A coated fibers incorporated within the hydrogel (right panel) after one day, seven days and 14 days of culture. Scale bars: Bright‐field: 1,000 µm and live/dead: 300 µm. (D) Cryo‐SEM images of GelAGE hydrogels with encapsulated glomerular co‐culture consisting of hMC, hGEC, and hPodocytes. Left picture: without cells, middle picture: with cells from the co‐culture spheroid after one day of cultivation, and right picture after seven days of cultivation. Encircled structures: cells. Arrows: Area altered by cells. Scale bar: 10 µm. ECM: extracellular matrix, GelAGE: allyl‐modified gelatin, hGEC: human glomerular endothelial cells, hMC: human mesangial cells, hPodocytes: immortalized human podocytes, VEGF‐A: vascular endothelial growth factor‐A. Experiments with GelAGE were performed more than three times.

After one day of maturation, glomerular spheroids were mixed into the hydrogel suspension. GelAGE was cured using UV radiation (Figure 6A). The short UV exposure for cross‐linking did not affect the viability of cells (Figure S10).

Mono‐cultures of human mesangial cells and human glomerular endothelial cells in GelAGE showed moderate survival, but podocytes in mono‐culture revealed cell death after several days in GelAGE (Figure S11). In contrast, glomerular co‐cultures could be kept in GelAGE for up to 14 days before they were stained with phalloidin (Figure 6B). Starting from day 1 in culture, cells spread out of the spheroids and formed cellular networks (Figure 6B; Videos S2–S7). As for spider silk, we hypothesized that VEGF‐A might increase cell viability and cell interaction within GelAGE. Therefore, we compared the cell survival of glomerular spheroids embedded in GelAGE, added with VEGF‐A‐coated fibers, to standard GelAGE hydrogels. However, VEGF‐A‐coated fibers did not improve the viability of cells in GelAGE (Figure 6C). To further characterize the hydrogel, we examined its pore structure using cryogenic scanning electron microscopy (cryo‐SEM). GelAGE hydrogels without (Figure 6D left) and with incorporated glomerular co‐culture (Figure 6D middle and right) were compared after one and seven days of incubation. Over time, cells spread throughout the gel, divided or form cell connections. Thus, after seven days, the areas surrounding the cells showed channel‐like structures. The encircled areas in Figure 6D show the perimeters of cells, whereas arrows point at the surrounding, indicating the disruption of the homogenously distributed pores.

For a more detailed display of the cell survival of co‐culture spheroids, embedded in GelAGE, a Calcein‐Hoechst staining was performed over a period of 14 days (Figure S12). The round shape of the spheroids changed over the two‐week timespan into a widely spread cell arrangement.

### 2.7 Mechanical Characteristics of GelAGE Hydrogel with Encapsulated Glomerular Co‐Culture Spheroids

Similar as done for spider silk‐based gels, we investigated how glomerular cells interact with GelAGE and whether the gel's properties change over time. First, cyclic loading tests were performed without cells and determined the complex mechanical properties, including nonlinearity, hysteresis, and conditioning (Figure S13). While an increase in the nominal stresses, nonlinearity, hysteresis, and conditioning from day 0 to day 1 was visible for all loading modes, the material properties remained similar on day 7 and day 14.

**FIGURE 7:**
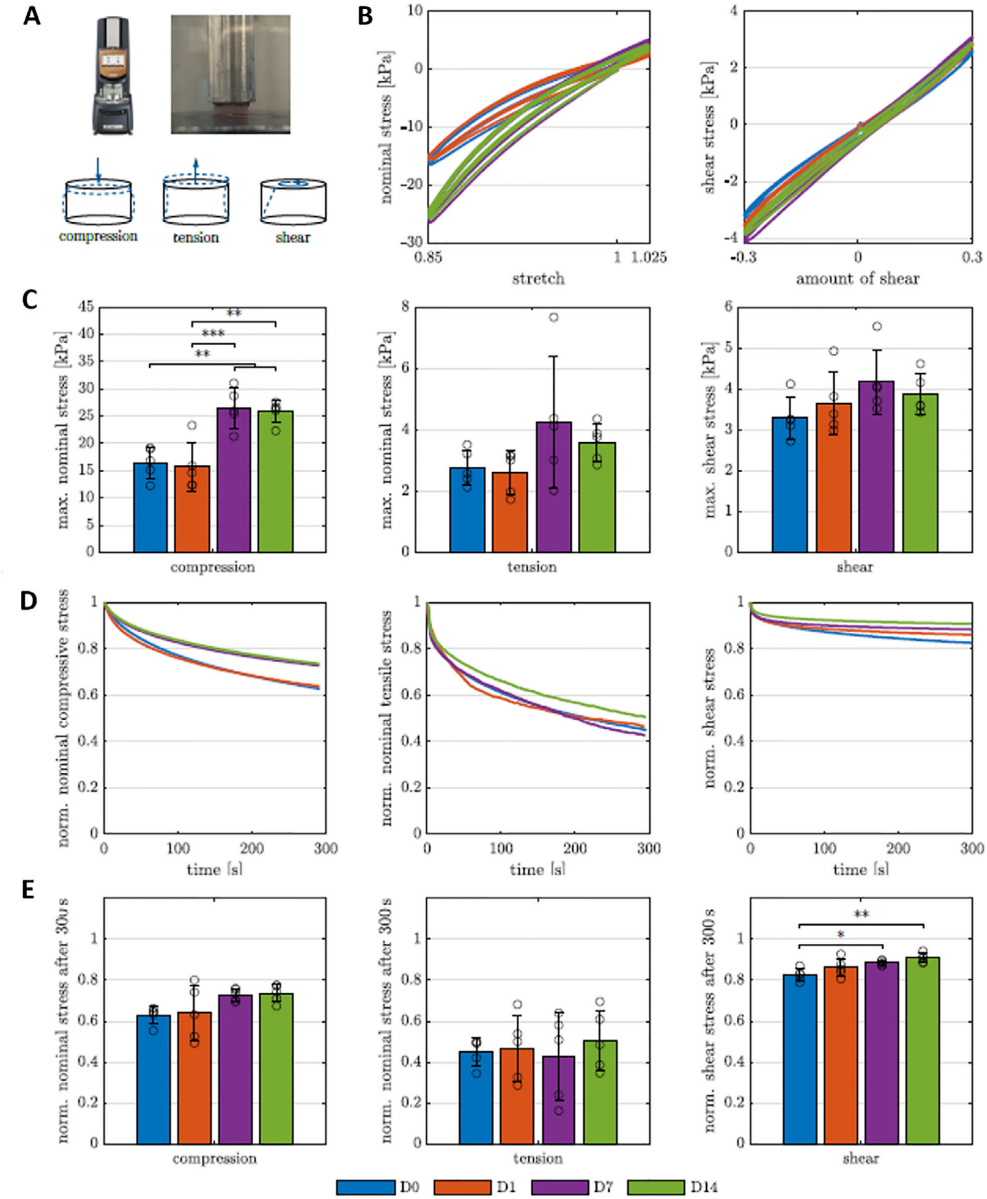
Mechanical characterization of GelAGE hydrogel with encapsulated glomerular co‐culture. (A) Testing setup: Rheometer to perform multi‐modal mechanical testing of glued cylindrical GelAGE samples in compression, tension, and torsional shear under large strains. (B) Cyclic loading behavior of cell‐laden GelAGE hydrogels (*n* = 5 for each time point) during cyclic compression‐tension up to a maximum strain of 15% in compression, 2.5% in tension (left), and during cyclic shear up to a maximum shear of 30% in torsional shear (right) over 14 days of cultivation. (C) Corresponding average maximum nominal stresses in compression (left), tension (center), and torsional shear (right) over 14 days of cultivation. (D) Average normalized stress relaxation behavior of GelAGE hydrogels with encapsulated glomerular co‐culture consisting of hMC, hGEC and hPodoytes at a maximum strain of 15% in compression (left), 2.5% in tension (center), and at a maximum shear of 30% in torsional shear (right) over 14 days of cultivation. (E) Corresponding average normalized stress after 300s relaxation in compression (left), tension (center), and torsional shear (right) over 14 days of cultivation. Significances were calculated using one‐way ANOVA if all samples were normally distributed and Kruskal‐Wallis tests otherwise, followed by Tukey‐Kramer tests for multiple comparisons. Significance values: ^*^ *p* < 0.05, ^**^ *p* < 0.01, ^***^ *p* < 0.001.

Cyclic loading and stress relaxation tests under compression, tension, and torsional shear were performed on GelAGE hydrogels containing encapsulated glomerular spheroids (Figure 7A), and key mechanical characteristics, including nonlinearity, hysteresis, conditioning, and stress relaxation, were systematically evaluated. From day 7 onward, glomerular cell‐containing GelAGE hydrogels showed a more pronounced nonlinear behavior with increasing nominal stresses during cyclic loading but similar hysteretic behavior and (pre)conditioning effects compared to day 0/day 1. The hysteretic behavior was evaluated by the hysteresis area, defined as the enclosed area between the loading and unloading curve of the stress‐stretch or stress‐shear response during each cycle, which quantifies the corresponding energy dissipation of each cycle. As the cell network matured over time, the nominal stresses significantly increased in compression, and slightly, but not significantly, in tension and torsional shear from day 0/day 1 to day 7/day 14 (Figure 7B,C). No further changes in the mechanical properties from day 7 to 14 could most likely be attributed to a strongly developed cell network after seven days of incubation (c.f. Figure 6B).

During stress relaxation tests, GelAGE hydrogels containing glomerular cells exhibited characteristic viscoelastic behavior, relaxing up to 37% under compression, 55% under tension, and 17% under torsional shear on day 0, without reaching equilibrium stress values within 300 s. Over time, a general trend of decreasing viscous effects was observed across all loading modes from day 0 to day 14, with the exception of tension between day 1 and day 7 (Figure 7D,E). This reduction in viscous response likely reflects the maturation of cellular networks within the polymer matrix (Figure 6B). The exception in tension on day 7 may be explained by deviations in the stress relaxation data for that time point (Figure 7E, center).

## 3 Discussion

Our understanding of many cell‐based processes derives from experiments performed on flat, stiff materials such as polystyrene and glass in 2D. Cells cultured under these conditions show unphysiological shape and polarization, altered response to pharmaceutical reagents, and loss of differentiated phenotype [5, 23]. Culture models that better mimic the biological milieu are needed to gain experimental results of translational meaning *in vitro*. Especially in the glomerulus, where multiple cells interact directly and indirectly for proper function, 2D mono‐cultures do not recapitulate the situation *in vivo*. Furthermore, scaling‐up opportunities are needed but not possible without scaffolds supporting the extracellular stability and cohesion of several cellular subunits. Hydrogels were shown to control mechanical, compositional, and structural cues and, thus, more accurately represent features of native tissues in cell culture [24].

This study aimed to find a suitable hydrogel formulation to support long‐term glomerular 3D co‐cultures. Many factors should be considered when selecting a hydrogel. These include handling of the gel, adhesivity to cells, stability in culture, cell survival of all cell types involved, and behavior for the specific cell types of interest and biophysical properties. Since most other studies only test their matrices on one or two cell lines, here we highlight that it is of great importance to match all the needs of every cell type.

We tested five different hydrogels for glomerular spheroid culture, for the first time, to identify those most suitable for long‐term culture in a 3D environment with an ECM‐like structure.

Matrigel has been used as a scaffold material in a wide variety of experimental setups over the last few years [25, 26]. It is a promising hydrogel for glomerular co‐culture as it contains collagen and laminin, which are also found in the glomerular ECM and glomerular basement membrane [27]. Matrigel is very soft (0.45 kPa) [28] and, therefore, allowed human mesangial cells to spread. However, it was very unstable, and the gel dissolved in the cell culture medium only after a few days and, therefore, was considered not suitable for glomerular co‐culture models.

Even though ADA‐GEL was stable with precise mTG concentrations for mesangial cells, this gel showed huge batch‐to‐batch variations, was not transparent enough, and therefore caused background for imaging and revealed some toxicity to cells. Furthermore, the culture of glomerular endothelial cells and podocytes was not possible as ADA‐GELs kept dissolving with these cells.

Human glomerular mesangial cells and human glomerular endothelial cells survived and spread in a fibrin‐based gel. However, this gel also degraded very fast (within 7 days), and increasing concentrations of aprotinin to stabilize the gel affected cell survival. Imaging through the gel was difficult due to its opaque appearance.

Biocompatibility, biodegradability, and potential for processing in aqueous solution under ambient conditions render silk‐based materials promising scaffolds for tissue engineering [29]. For hydrogel generation, self‐assembly of ß‐sheet‐rich spider silk nanofibrils nucleation‐aggregation is followed by concentration‐dependent gelation [30, 31]. Large quantities of engineered spider silk proteins for hydrogel formation are possible with recombinantly produced spider silk protein eADF4(C16) (a recombinant protein based on the consensus sequence of *Araneus diadematus* Fibroin 4 (ADF4)) [32]. Steiner et al. were able to show the effective role of recombinant spider silk fibrous scaffolds concerning their vascularization in an arteriovenous loop model [19]. Therefore, spider silk seemed to be a good choice for glomerular co‐culture models as it showed good biocompatibility, stability, and modifiability. Podocytes, glomerular endothelial cells, and mesangial cells all showed good survival in this gel in 3D co‐culture. Light‐sheet microscopy was able to visualize cell sprouting out of spheroids seeded in spider silk. Furthermore, the mechanical characterization of spider silk hydrogels with glomerular spheroids revealed changes in the multimodal mechanical response including a pronounced nonlinearity, an increase in the maximum nominal stresses in compression and torsional shear after 1 day of incubation, and slightly less pronounced viscoelastic effects due to maturation of the cells after 1 day of incubation.

However, the disadvantages of spider silk include the fact that it requires great know‐how and that crucial steps can only be controlled with a certain level of expertise. This material is very time‐ and temperature‐critical during the fabrication of hydrogels. However, with further adjustments to the handling process, excellent sample quality could be achieved. Other hydrogels may be more suitable for quick and easy handling. Nevertheless, this matrix has good properties for glomerular cells.

Gelatin has previously been successfully modified with allyl functional groups (GelAGE) and cross‐linked via UV radiation [21, 33]. Nevertheless, GelAGE has not been exhaustively studied for tissue engineering and 3D cell culture applications. Glomerular spheroids consisting of podocytes, glomerular endothelial cells, and mesangial cells survived for up to 14 days. Therefore, cell survival for all three glomerular cell types was the longest in GelAGE. Moreover, this gel was stable and clear enough for imaging analyses. Not only confocal microscopy but also multiphoton microscopy was able to visualize cells within the gel, which spread out of the spheroids. To the best of the authors’ knowledge, this study provides the first systematic, in‐depth investigations of the influence of glomerular spheroids on the complex mechanical properties of GelAGE hydrogels and confirmed changes in the multimodal mechanical response including a pronounced nonlinearity, an increase in the maximum nominal stresses and slightly less pronounced viscoelastic effects due to maturation of the cells after 7 days of incubation. Nevertheless, the trend of increasing maximum nominal stresses of GelAGE hydrogels without cells during unconfined compression from day 0 to day 14 is in accordance with our previous study for GelAGE hydrogels (2.75% w/v GelAGE + 2.75% w/v PEG‐4‐SH) with a low GelAGE concentration [33].

## 4 Conclusion

This is the first study investigating five different hydrogels for glomerular 3D co‐cultures composed of three different cell types, which were fabricated in larger scale. GelAGE was found to provide the best ECM for glomerular spheroids and allowed the culture cells to grow for up to 14 days. Confocal and multiphoton microscopy were able to visualize the cells within the gel, and mechanical characterization of GelAGE and spider silk hydrogels containing glomerular spheroids revealed viscoelastic effects of the hydrogel, which are important for cell spreading and proliferation within the matrix. Hence, GelAGE and spider silk might be a good choice for glomerular co‐culture models in the future.

## 5 Experimental Section/Methods

### 5.1 Cell Culture

Human mesangial cells and human glomerular endothelial cells (both purchased from Cell Systems, Kirkland, USA) were kept at 37°C and 5% CO_2_ (HERAcell vios 160i incubator, Thermo Scientific, Langenselbold, Germany), whereas human podocytes (kindly provided by Moin Saleem, Children's Hospital and Renal Unit and Bristol Renal, University of Bristol) were kept at 33°C and 5% CO_2_. For each type of cell, a particular medium was used. Human podocytes were maintained in medium consisting of RPMI 1640 medium (Gibco, ThermoFisher Scientific, Waltham, USA), 10% heat‐inactivated FCS (Pan Biotech, South American origin, Aidenbach, Germany), 1% penicillin‐streptomycin (Sigma Aldrich, St. Louis, USA), and 0.1% insulin‐transferrin‐selenium (ITS, Gibco, Grand Island, USA). Human mesangial cells were kept in the same medium but without ITS. For human glomerular endothelial cells, VascuLife EnGS‐Mv Microvascular Endothelial Kit (Lifeline Cell Technology, San Diego, USA) was used. All three media were mixed for the co‐culture medium, which is referred to as “PEM” medium in the following. An overview of media composition for the different cell types can be found in Table 1.

**TABLE 1:** Overview of media composition for the different cell types.

Cell type	Medium composition	Supplier/Notes
Human podocytes (hPodo)	‐ RPMI 1640 medium (500 mL) ‐ heat‐inactivated fetal calf serum (FCS; 10%) ‐ Penicillin‐Streptomycin (1%) ‐ Insulin‐Transferrin‐Selenium (ITS; 0.1%)	Gibco (USA), Pan Biotech (South America origin, Germany), Sigma Aldrich (USA)
Human mesangial cells (hMC)	‐ RPMI 1640 medium (500 mL) ‐ heat‐inactivated fetal calf serum (FCS; 10%) ‐ Penicillin‐Streptomycin (1%)	Same as podocytes, without ITS
Human glomerular endothelial cells (hGEC)	‐ VascuLife EnGS‐Mv Microvascular Endothelial Medium Kit, consisting of:	Lifeline Cell Technology (San Diego, USA)
	• VascuLife basal medium (500 mL) Ascorbic Acid (50 µg/mL) • Hydrocortisone Hemisuccinate (1 µg/mL) • L‐Glutamine (10 mM) • Recombinant Human EGF (5 ng/mL) • Heparin Sulfate (0.75 U/mL) • rh EnGS (0.2%) • FBS LifeFactor (5%) • Antimicrobial Supplement: Gentamicin (30 µg/mL), Amphotericin B (15 µg/mL)	
PEM medium (co‐culture medium)	‐ A mixture of the three media above	Used for co‐culturing podocytes, endothelial cells, and mesangial cells

### 5.2 Spheroid Fabrication

For the co‐culture of spheroids, 3,000 podocytes, 2,000 glomerular endothelial cells, and 1,000 mesangial cells were used per spheroid. For mono‐culture spheroids, 6,000 cells of the respective cell types were used. There are multiple ways to produce spheroids, but for this work, two methods were used depending on the spheroid yield needed. Fabrication of spheroids in agarose micro‐wells was best for large amounts of spheroids, where the cell suspension was distributed evenly by gravity to form spheroids. The agarose micro‐wells were made of 2.5% sterile agarose (VWR Life Science, Leuven, Belgium). Agarose was poured on top of negative molds (3D printed with a digital light processing printer (Prusa SLS1 speed, Joseph Prusa Research, Czech Republic)) and left to harden for 20 min. Then, UV light for sterilization was applied for 30 min. Final agarose micro‐wells were cut out (Boehm hole puncher, La Fouillouse, France) and transferred to a 6‐well plate (Sarstedt, Nümbrecht, Germany) where cell suspension and medium were added. After one day of cultivation, spheroids were stable enough for further experiments. For smaller numbers of spheroids, Nunclon Sphera‐treated 96 U‐bottom well plates (Thermo Scientific, Waltham, USA) were used. First, the PEM medium was placed in each well, followed by the cell suspension. Similar to the aforementioned method, spheroids were also formed and ‘stable‐to‐use’ after one day of cultivation.

### 5.3 VEGF‐A Coated Poly‐L‐Lactic Acid (PLLA) Fiber Production

The fibers were fabricated as previously described by Lamberger et al. [34] and made from PLLA material (Purasorb PL65, Corbion, Netherlands). The coating with VEGF‐A (Vascular Endothelial Growth Factor 165 human, Sigma Aldrich, St. Louis, USA) was done via polydopamine immobilization as published by Sun et al. [20], establishing a load of 100 ng of VEGF per mg of fibers. After coating, the fibers were washed and kept in PBS at −20°C until use. The coating efficiency on the fibers was determined by human VEGF‐165 ELISA Development Kit (ABST; PeproTech, Rocky Hill, USA), by detection of unbound VEGF‐A presence in the supernatant of the coating solution, which turned out to be minimal, confirming that 99.83 ng/mg of growth factors were indeed on the fibers (Figure S7). VEGF‐A coated PLLA fibers were incorporated either into the spheroids by mixing the fibers into the cell suspension before distributing to well plate/micro‐wells or into the hydrogel solution itself. Fiber concentrations were 0.5 µg/spheroid or 0.1 mg/mL final hydrogel gel volume. Loading efficiency and release of VEGF‐A from fibers were described before [20].

### 5.4 Cell/Spheroid Seeding into Hydrogels

Matured spheroids were collected in falcons and left to settle on the bottom. The medium was carefully removed. Spheroids or cell pellets from cell suspensions were then resuspended in corresponding hydrogel components without creating bubbles. Approximately 10–20 spheroids/hydrogels were used.

### 5.5 Live/Dead Staining for Cell Viability

Live/dead staining was performed with a commercially available live/dead kit (Invitrogen, Eugene, USA) to confirm cell viability in respective hydrogels. Calcein AM acted as a live cell indicator and BOBO‐3‐iodide as a dead cell indicator. The kit was performed according to the manufacturer's instructions. Only the incubation time was adapted to 30 min. For the dead‐cell control, samples were treated with 4% PFA for 45 min. Imaging was done with the Acquifer Imaging Machine (Luxendo, Heidelberg, Germany).

### 5.6 Calcein‐Hoechst Staining

Calcein‐Hoechst staining was done with purchasable Calcein AM dye (Invitrogen, Eugene, USA) and Hoechst 33342 (20 mm, Thermo Scientific, Rockford, USA). A 2 µm Calcein working solution was added to the samples together with Hoechst 33342 (1:1000). The incubation time was prolonged to 1 h at 37°C for hydrogel staining. Dead control samples were treated with 70% ethanol for 1 h. Imaging was done with a laser scanning confocal microscope (Leica DMI6000 inverted microscope, Leica Microsystems, Wetzlar, Germany).

### 5.7 Confocal Microscopy

Samples were fixed in 4% PFA (Carl Roth, Karlsruhe, Germany) for 15 min. Pre‐incubation solution (10% normal goat serum (Abcam, Cambridge, UK), 1% BSA (Karlsruhe, Germany), 0.5% Triton X‐100 (Carl Roth, Karlsruhe, Germany) in PBS) was added to the samples for one hour (RT, dark) to permeabilize and block unspecific binding sites. Afterward, WGA‐555 (Biotium, Fremont, USA) was diluted 1:100 in antibody diluent (3% normal goat serum and 1% BSA in PBS) and incubated for 1.5 h (RT, dark). Samples were washed thoroughly with PBS. Hoechst 33342 (Stock: 50 µg/mL) (Thermo Scientific, Rockford, USA) was used 1:200 in PBS for 5 min (RT, dark). Lastly, samples were washed before imaging with a laser scanning confocal microscope (Leica DMI6000 inverted confocal microscope, Leica Microsystems, Wetzlar, Germany).

### 5.8 Multiphoton Microscopy

Pre‐incubation solution containing Triton X‐100 was added to the samples for one hour (RT, dark). Samples were then washed with PBS. Phalloidin‐647 (Thermo Fisher, Eugene, USA) was diluted 1:200 in antibody diluent and incubated for 1.5 h (RT, dark). Samples were then thoroughly washed with PBS. Samples were imaged using a multiphoton microscope (Trim‐Scope II, LaVision BioTec, Bielefeld, Germany) coupled with a mode‐locked femtosecond‐pulsed Ti:Sa laser (Chameleon Vision II, Coherent, Santa Clara, USA; pulse frequency: 80 MHz). For measuring the glomerular cells embedded within a hydrogel, a water immersion objective (Leica HC Fluotar L 16X/0.60 IMM CORR VISIR, Leica Microsystems, Wetzlar, Germany) was used. The fluorescence signal was detected, utilizing photomultiplier tubes (H 7422–40 LV 5 m, Hamamatsu Photonics, Herrsching, Germany) which were equipped with dichroic mirrors (Chroma ET‐series, Chroma Technology Corporation, Bellow Falls, USA) and bandpass filters (Chroma T‐series, Chroma Technology Corporation, Bellow Falls, USA) for spectral separation. To obtain details of the 3D architecture of the cells embedded in the hydrogel, 3D image stacks were recorded (lateral pixel size: 0.6 µm, line scanning frequency: 1,000 Hz, step size axial direction: 0.6 µm; pixel dwell time: 0.7 µs, physical voxel size: 0.6 × 0.6 × 0.6 µm). Images were analyzed and compiled via ImageJ for 3D visualization.

### 5.9 Light Sheet Microscopy

Fixed and with pre‐incubation solution (see above) permeabilized samples were washed and stained with Phalloidin‐647 (Thermo Fisher, Eugene, USA, 1:100) and WGA‐555 (Biotium, Fremont, USA, 1:100) for 1.5 h (RT, dark). For imaging, a light sheet microscope (TruLive3D Imager, Luxendo, Bruker) was used.

### 5.10 Preparation of Matrigel

For working with Matrigel Basement Membrane Matrix, LDEV‐free (Corning, Bedford, USA), all steps were prepared on ice. Matrigel domes were prepared using drops of hydrogel containing 600 cells. Round coverslips (14 mm) were placed on the rims of two neighboring wells of a 12‐well plate (Sarstedt, Nümbrecht, Germany). One drop of Matrigel‐cell‐mixture was positioned in the middle of the coverslip and very carefully flipped so that the drop was hanging on the bottom side of the coverslip between the rims of the wells. This was left to dry for 8 min. After the formation of the dome shape, the coverslip was placed (with the sample side facing upward) in a 24‐well plate and left to fully solidify in the cell incubator for 30 min. Then medium was carefully added into the wells.

### 5.11 Preparation of ADA‐GEL

Gels with 2.5% (w/w) were prepared by mixing equal amounts of ADA and gelatin. For this, gelatin needed to be pre‐warmed at 37°C. The polydimethylsiloxane (PDMS) molds (height = 2 mm, diameter = 6 mm) were filled with ADA‐GEL cell mixture and then placed in Petri dishes so that the molds could be doused with a 0.1 m CaCl_2_ cross‐linker solution. After 10 min, the hydrogels were removed from the CaCl_2_ solution and carefully removed from the molds to transfer them into well plates containing cell culture medium. After initial experiments, ADA‐GEL needed to be modified for better gel stability over time. Hence, microbial transglutaminase (mTG) was introduced for the prolongation of hydrogel durability in culture conditions. The protease inhibitor was used in the ADA‐GEL mixture (25 µL/1 g hydrogel final volume of 40% mTG solution) as well as in the CaCl_2_ cross‐linking solution (2.5 mL of 20% mTG solution/dish). The cross‐linking solution was exchanged for cell culture medium after 10 min.

### 5.12 Preparation of Fibrin Gel

A commercially available TISSEEL (fibrin sealant) Kit (Baxter, Unterschleißheim, Germany) was taken apart to adjust the concentrations of the individual components. Here, a high and a low fibrinogen concentration were compared to find the best composition for cells and the experimental setup. Fibrinogen was diluted with PBS to a concentration of 22.75 mg/mL (high concentration) and 15 mg/mL (low concentration), and thrombin was used at a concentration of 50 IU/mL for both approaches. Respective cells (50,000 cells/hydrogel) were harvested, and the cell pellet was resuspended in the thrombin component. Both components were then mixed in equal parts, and the mixture was placed into PDMS molds. For fibrinogen cleavage and subsequent polymerization of fibrin, the gels were incubated in a cell culture incubator for at least 40 min. Finally, gels were transferred into the cell medium.

### 5.13 Modification with Aprotinin

As degradation occurred quickly with fibrin hydrogels, a protease inhibitor within the blood coagulation cascade called aprotinin (Roche Diagnostics, Mannheim, Germany) was added to the cell culture medium (20 µg/mL) to prolong the lifetime of the hydrogels. Medium change was done every day with freshly prepared aprotinin‐supplemented medium.

### 5.14 Preparation of eADF4(C16)‐RGD Hydrogels

The synthesis and production of the eADF4(C16)‐RGD protein were described before [35]. For the preparation of eADF4(C16)‐RGD hydrogels, the lyophilized protein was first dissolved at 15 mg/mL in 6 m guanidinium thiocyanate (Carl Roth GmbH, Karlsruhe, Germany) for 60 min in an overhead shaker at room temperature. The denatured protein was then dialyzed against an excess of 10 mm TRIS‐HCl (pH 7.5) buffer using dialysis tubes with a MWCO of 6–8 kDa (Spectra/Por MWCO, Repligen, San Diego, USA) over 16 h, with the last step being carried out overnight. The protein solution was then collected, centrifuged (17,000 g, 20 min), and sterile‐filtered (pore size = 0.20 µm, Sarstedt AG & Co. KG, Nümbrecht, Germany). To increase the protein concentration, the solution was dialyzed against 25% (wt) PEG (40 000 kDa, SERVA, Heidelberg, Germany) for one to two hours until it reached a concentration of at least 35.3 mg/mL. The eADF4(C16)‐RGD stock was then pre‐cross‐linked in a water bath at 37°C for 1–1.5 h to allow for rapid gelation after encapsulation and was stored on ice until needed. The silk solution was diluted with cell culture medium and sterile 10 mm TRIS‐HCl buffer to a final concentration of 30 mg/mL eADF4(C16)‐RGD containing a 15% (v/v) medium.

### 5.15 Spheroids in Spider Silk Hydrogels

Previously fabricated spheroids were taken up in PEM medium in 15% of the final gel volume. The freshly prepared spider silk solution was mixed with the cells in the medium. The mixture was distributed into the PDMS molds and then left to self‐assemble in the incubator at 37°C (5% CO_2_). After solidification, 3% spider silk hydrogels were removed from molds and transferred into well plates with PEM medium. Composite hydrogels with a collagen component were made with 1% spider silk and mixed at a ratio of five parts spider silk solution to four parts collagen IV (Advanced Biomatrix, San Diego, USA).

### 5.16 Synthesis of Allyl‐Modified Gelatin

GelAGE, based on type A porcine gelatin (Sigma Aldrich, Darmstadt, Germany), was dissolved in deionized water (10%) at 37°C over two hours. Two molar NaOH solution and AGE were mixed into the gelatin mixture at 65°C for an additional two hours. The reaction takes place in an alkaline milieu, which enables the deprotonation of the amino acids of the gelatin. During nucleophilic substitution, these react with the carbon site of the AGE ring, resulting in allyl‐modified gelatin. The GelAGE G2LH was then dialyzed (Spectra/Por 6, Fisher Scientific, Hampton, USA, molecular weight cut‐off (MWCO) = 1 kDa) against deionized water and subsequently lyophilized.

To determine the Degree of Modification (DoM), proton nuclear magnetic resonance spectroscopy (^1^H‐NMR, Bruker Biospin 400 MHz spectrometer) was done with deuterium hydroxide (D_2_O) as solvent (Figure S9). The reference signal for AGE modification was phenylalanine (peaks at δ = 7.30–7.10 ppm) and calibrated to 5 protons. To assess the DoM of the functionalized gelatin, the integral of the allyl group protons was compared to that of the phenylalanine signals. The calculation of the DoM was done as described in Cianciosi et al. [36].

### 5.17 Preparation of GelAGE

Lyophilized GelAGE (3% w/v) was dissolved in PBS together with commercially available PEG‐4‐SH (3.9% w/v, JenKem Technologies, Plano, USA). The solution was UV sterilized (UV lamp, Vilber Lourmat VL‐6.LC 365/254 nm, Marne‐la‐Vallée, France) for 20 min at λ = 254 nm setting. GelAGE‐PEG‐4‐SH solution, previously fabricated spheroids, and lithium phenyl‐2, 4, 6‐trimethylbenzoylphosphinate (LAP, 0.2%, Sigma Aldrich) were mixed. LAP functioned as a photo initiator in the polymerization process of GelAGE, where PEG‐4‐SH acted as cross‐linker. The suspension was pipetted into PDMS molds and cross‐linked at λ = 365 nm (distance between the sample and UV lamp = 4 cm) for 50 s. Hydrogels were transferred into cell culture well plates with PEM medium and cultured in an incubator at 37°C and 5% CO_2_.

### 5.18 Cryo‐SEM for Structural Analysis

Hydrogels were placed between two metal plates (diameter of 3 mm) and quickly frozen in a nitrogen slush. For transferring steps between devices, an EM VCT100 cryo‐shuttle (−156°C, Leica Microsystems) was used. To produce the imaging surface, the sample between the metal plates was broken in half to reveal the inside structure. This was done by chipping off the upper part of the clamped sample under a high vacuum atmosphere in a Sputter Coater machine (ACE 400, Leica Microsystems, Wetzlar, Germany) at –85°C for 15 min, where it was also subsequently coated with a 2.5 nm thick layer of platinum. Scanning electron microscopy (Crossbeam 340, Zeiss, Oberkochen, Germany) in combination with an Everhart‐Thornley‐SE‐detector (Zeiss, Oberkochen, Germany) revealed the overall structure at 1,000 X and pore structure at 5,000 X magnification (an acceleration voltage of 8 kV was used). Images were analyzed with ImageJ software (version 1.53t, Wayne Rasband and contributors, National Institute of Health, Bethesda, USA).

### 5.19 Complex Mechanical Characterization

The Discovery Hybrid Rheometer HR 30 from TA instruments (New Castle, Delaware, USA) was used to perform multimodal mechanical tests in compression, tension, and torsional shear, summarized in Table 2, of cell‐laden GelAGE hydrogels on days 0, 1, 7, and 14. For soft spider silk hydrogels, we adapted the protocol and excluded testing in tension. After the calibration of the rheometer, an 8 mm circular piece of fine sandpaper with a grain size of 180 µm was glued to the upper and lower specimen holders to improve the adhesion between the sample and the smooth specimen holders. We chose a stainless‐steel plate with a diameter of 8 mm for the upper and an anodized aluminum plate with a diameter of 40 mm for the lower specimen holder. Afterward, a thin layer of cyanoacrylate adhesive, Pattex superglue ultra‐gel (Henkel AG & Co. KGaA, Düsseldorf, Germany), was added to the upper specimen holder. The sample was placed on a spatula and glued in the center of the upper geometry. Finally, a thin layer of the same adhesive was added to the lower specimen holder, the transparent immersion cup was inserted, and the upper specimen holder with the attached sample was slowly lowered down until full contact between the sample and the lower specimen holder was visually confirmed. After a waiting time of 60–90 s for the adhesive to dry, the sample was immersed in a PEM medium bath to ensure hydration during testing.

**TABLE 2:** Multimodal testing protocol for the Discovery Hybrid Rheometer HR 30 from TA instruments.

Experimental test	Loading mode	Stretch/Amount of shear	Strain rate	Holding time
Cyclic loading	Compression and tension	0.85 and 1.025	0.01/s	
Stress relaxation	Compression	0.85	0.025/s	300 s
Stress relaxation	Tension	1.025	0.025/s	300 s
Cyclic loading	Torsional shear	0.30	0.1667 rad/s	
Stress relaxation	Torsional shear	0.30	0.1 s (rise time)	300 s

### 5.20 Graphical Illustrations

Schematic illustrations of respective hydrogel fabrication with encapsulated glomerular cells were created with BioRender.

Created in BioRender. Müller‐Deile, P. (2025) https://BioRender.com/t34p977

Created in BioRender. Müller‐Deile, P. (2025) https://BioRender.com/y64r248

Created in BioRender. Müller‐Deile, P. (2025) https://BioRender.com/a88n167

Created in BioRender. Müller‐Deile, P. (2025) https://BioRender.com/k82p154

Created in BioRender. Müller‐Deile, P. (2025) https://BioRender.com/n74s642

Created in BioRender. Müller‐Deile, P. (2025) https://BioRender.com/i35o727

## Funding

This research was funded by Deutsche Forschungsgemeinschaft (DFG: German Research Foundation, project number 506565062) and awarded to JMD and DS. We gratefully acknowledge the support of the DFG project number 326998133 – TRR 225 (subprojects A01, B09, C01, C06, Z02).

## Conflicts of Interest

The authors declare no conflicts of interest.

## Supporting information




**Supporting File 1**: mabi70166‐sup‐0001‐SuppMat.docx.

**Supporting File 2**: mabi70166‐sup‐0002‐VideoS1.avi.

**Supporting File 3**: mabi70166‐sup‐0003‐VideoS2.avi.

**Supporting File 4**: mabi70166‐sup‐0004‐VideoS3.avi.

**Supporting File 5**: mabi70166‐sup‐0005‐VideoS4.avi.

**Supporting File 6**: mabi70166‐sup‐0005‐VideoS4.avi.

**Supporting File 7**: mabi70166‐sup‐0007‐VideoS6.avi.

**Supporting File 8**: mabi70166‐sup‐0008‐VideoS7.avi.

## Data Availability

The data that support the findings of this study are available from the corresponding author upon reasonable request.
