# Supplementary material for: Biocompatibility of Hydrogels for Glomerular 3D Co‐Culture: A Comparative Analysis
Source: Macromol Biosci. 2026 Feb 24;26(2):e00460. doi: 10.1002/mabi.202500460 (PMC12931831; doi:10.1002/mabi.202500460)
Supplement: Supplementary file 1 — Supporting File 1: mabi70166‐sup‐0001‐SuppMat.docx. [file MABI-26-e00460-s007.docx]

Supporting Information

Title: Biocompatibility of Hydrogels for Glomerular 3D Co-Culture: A Comparative Analysis

*Julia Eichermüller^1^, Jessica Faber², Xuen Ng^3^, Camilla Mussoni^4^, Julian Bauer^5^, Jonas Röder^6^, Alessandro Cianciosi^4^, Philipp Stahlhut^4^, Tomasz Jungst^4^, Jürgen Groll^4^, Dominik Steiner^7^, Thomas Scheibel^3^, Oliver Friedrich^5^, Taufiq Ahmad^4^, Aldo R. Boccaccini^6^, Silvia Budday^2^, and Janina Müller-Deile^1*^*

**Supplementary Figures**

| **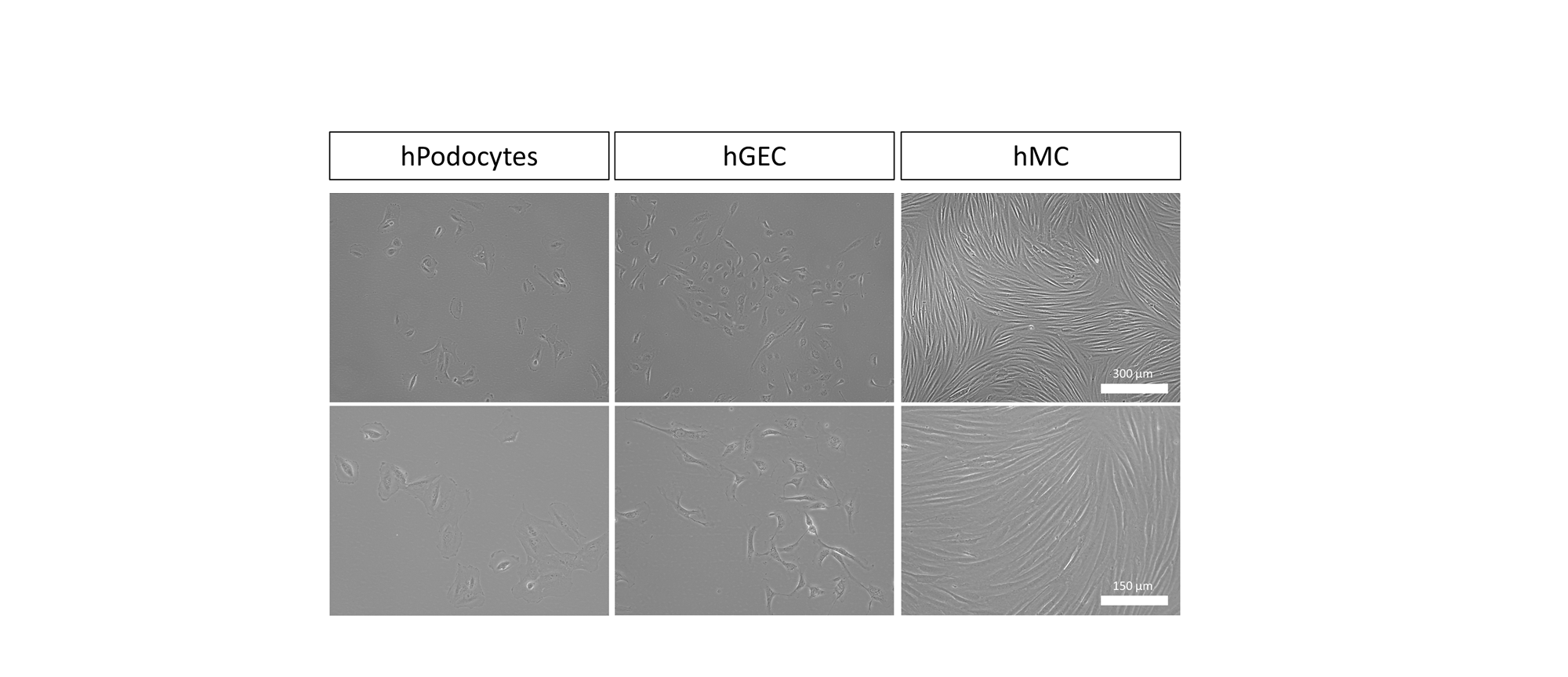** |
| --- |
| ***Figure S1: Mono culture of hPodocytes, hGECs and hMC in monoculture on plastic dishes.*** |
| Bright-field images of the cells are given in two different magnifications. The three different glomerular cell types possess different morphology. |
| hPodocytes: immortalized human podocytes, hGEC: human glomerular endothelial cells, hMC: human mesangial cells. |

**
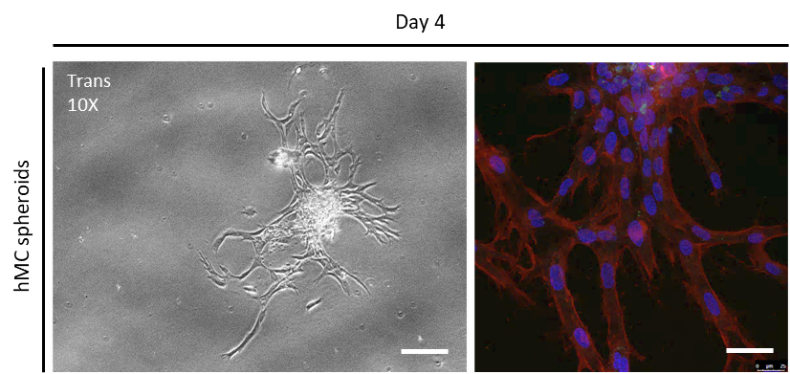
**

***Figure S2: Self-forming spheroids of human mesangial cells in Matrigel.***

Left: Bright-field microscopy of Matrigel shows self-formation of 3D mono-culture spheroids of hMCs after 4 days of culture. Scale bar: 300 µm. Right: Confocal microscopy of 3D hMC spheroids embedded in Matrigel domes after four days depicts cell elongation and cell-cell contacts. Nuclei were stained with Hoechst 33342 (blue), and cell surface marker WGA (red). Scale bar: 50 µm.

hMC: human mesangial cells.


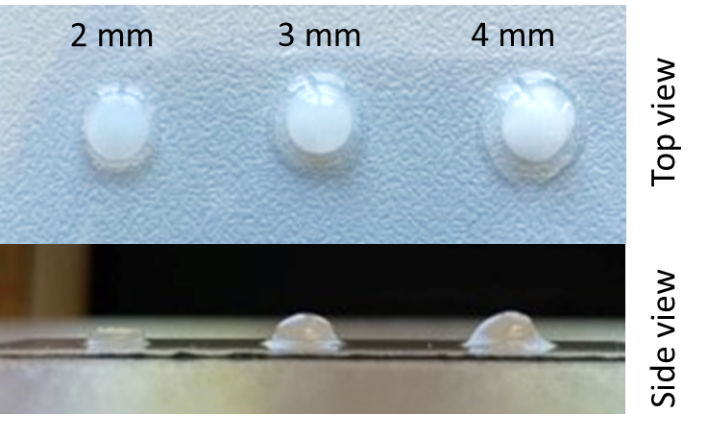


**Figure S3:** ***Macroscopic pictures of ADA-GEL***.

Macroscopic pictures of ADA-GEL from top view and side view show opaque appearance that does not allow clear bright field imaging of cells within the gel.

| **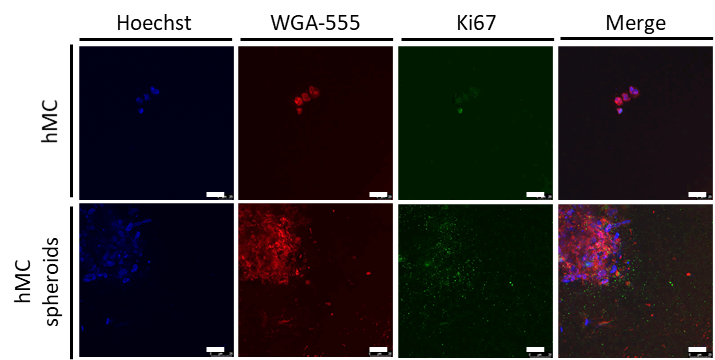**  **Figure S4:** ***Mesangial cells in ADA-GEL.***  Morphology of hMCs using confocal microscopy (upper panel: 2D mono-culture, lower panel: 3D mono-culture, blue: Hoechst 33342, red: WGA, green: Ki-67, right: merge). Scale bar: 50 µm.  ADA-GEL: alginate dialdehyde-gelatin, ECM: extracellular matrix, hMC: human mesangial cells, WGA: wheat germ agglutinin.  **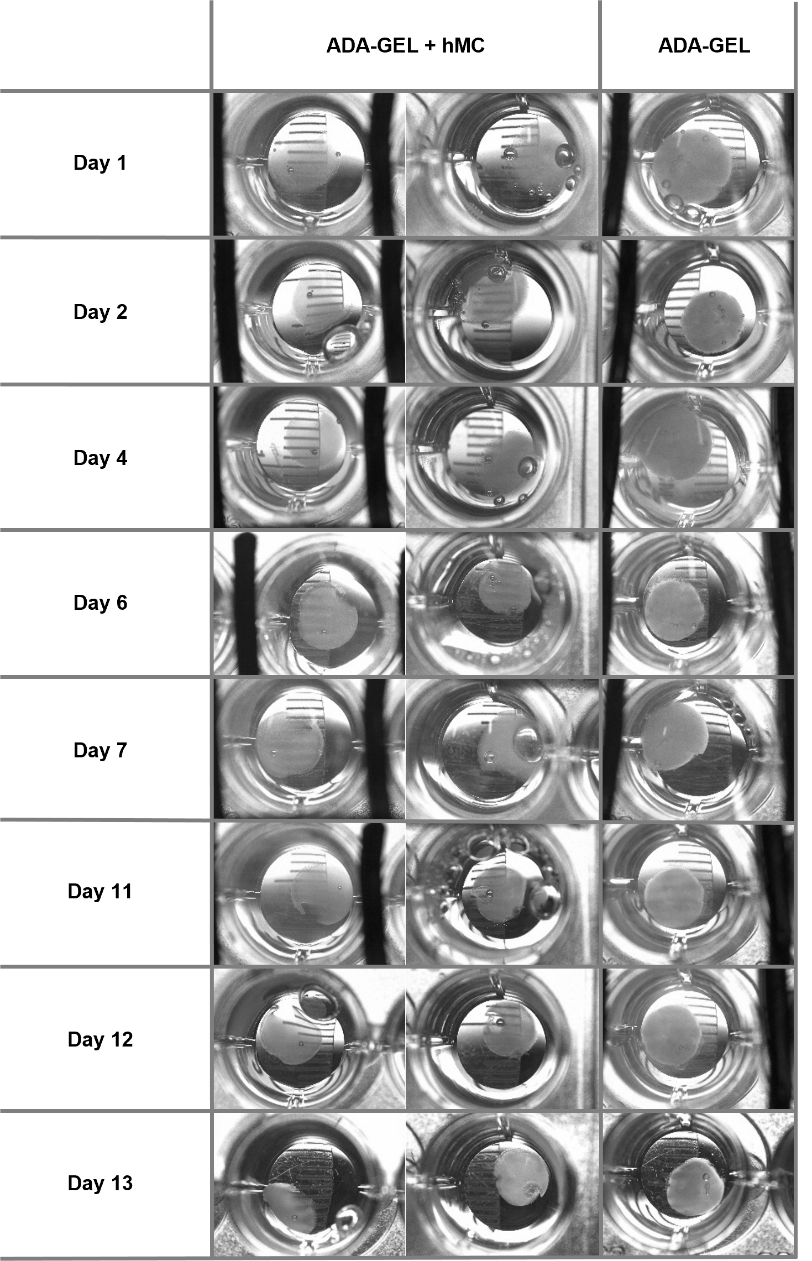** |
| --- |
| ***Figure S5: ADA-GEL degradation over time.*** |
| ADA-GEL fabricated with the addition of CaCl_2_ and mTG was incubated for 13 days either with hMC cells or without cells to compare gel stability. |
| ADA-GEL: alginate dialdehyde-gelatin, hMC: human mesangial cells, mTG: microbial transglutaminase, CaCl_2_: Calcium chloride |

| **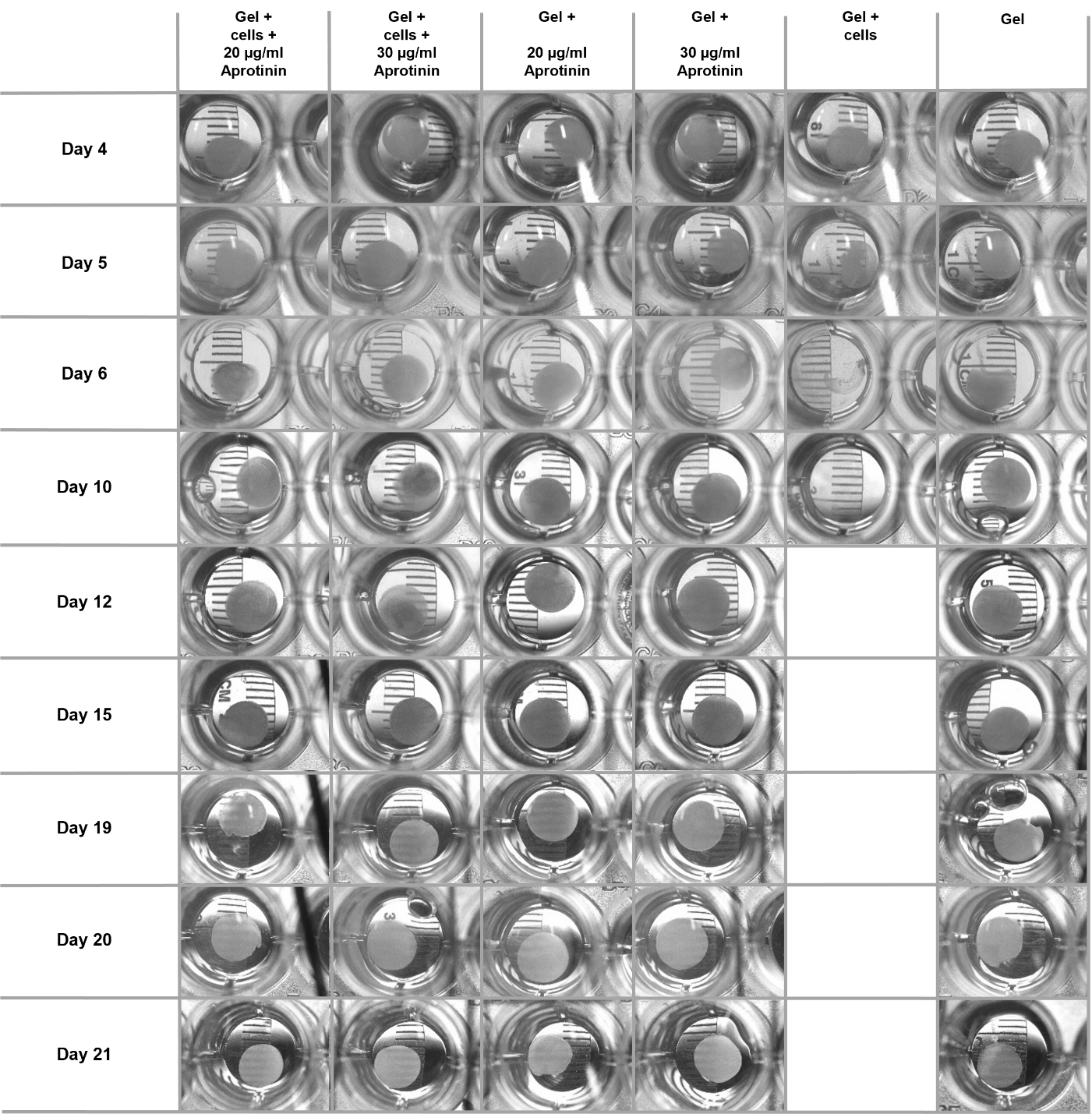** |
| --- |
| ***Figure S6: Fibrin hydrogel degradation over time.*** |
| Fibrin hydrogels with and without embedded cells were modified with two different aprotinin concentrations (20 µg/ml and 30 µg/ml) and compared to fibrin gels with only the addition of cells, as well as fibrin gels alone to distinguish degradation over 21 days. Fields lacking images correspond to areas where the gel had already undergone complete degradation. |

| 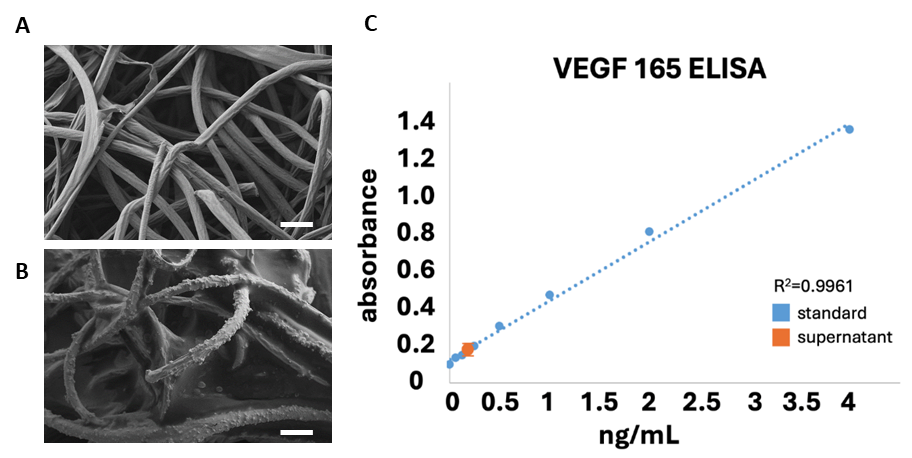 |  |
| --- | --- |
| **B** |  |
| **Figure S7: *Determination of binding efficiency of VEGF-coating on PLLA fibers by ELISA.*** | |
| A: PLLA fibers visualized by Cryo-SEM imaging. Scale bar: 2 µm.  B: PLLA fibers coated with VEGF visualized by Cryo-SEM imaging. Scale bar: 2 µm.  C: ELISA measurements of the remaining VEGF in the supernatant after incubation with the fibers. An average concentration of 0.17 ± 0.012 ng/ml was detected in the supernatant. Indicating that >99% of the VEGF was immobilized on the fibers, confirming efficient functionalization. | |
| VEGF: Vascular endothelial growth factor, PLLA: poly-L-lactic acid, ELISA: Enzyme-linked Immunosorbent Assay | |


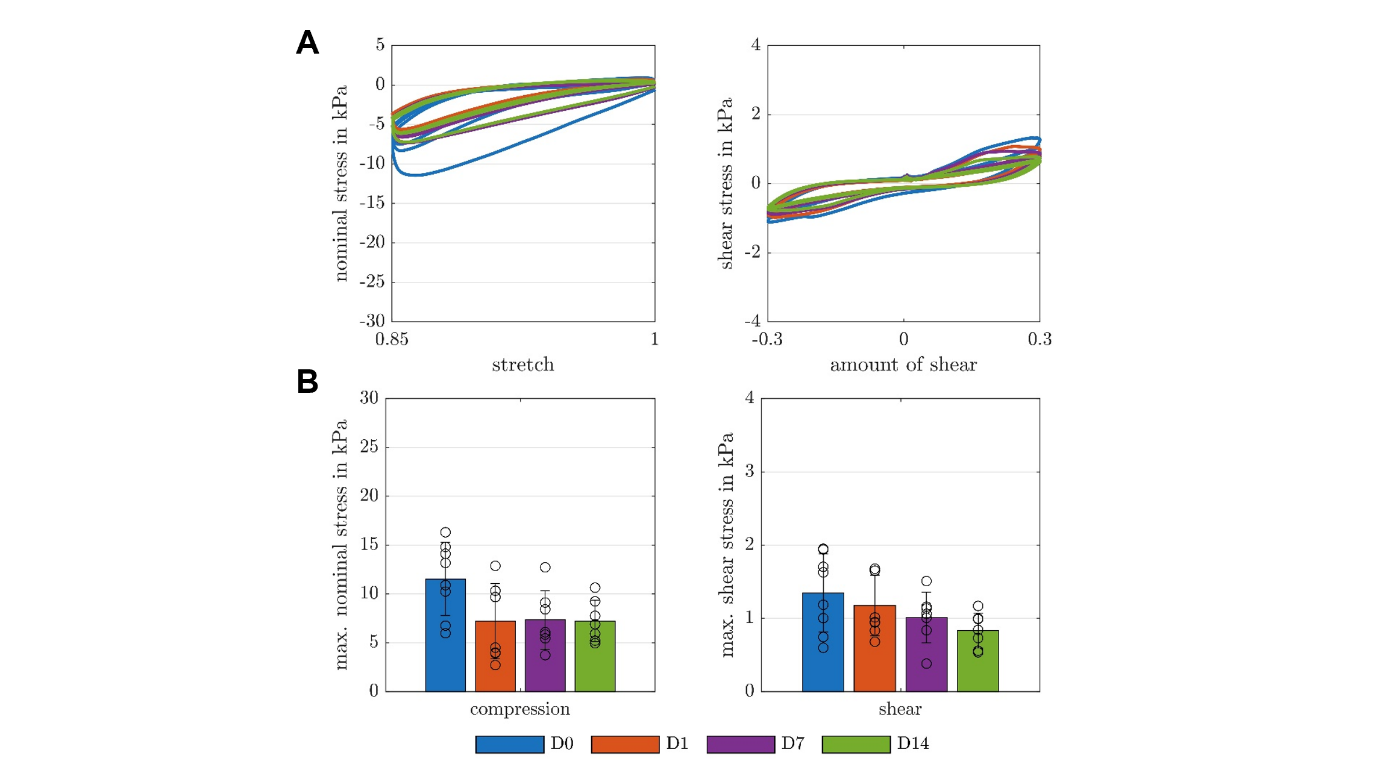


**Figure S8**: ***Mechanical characterization of spider silk hydrogel without spheroids***

A: Cyclic loading behavior of spider silk hydrogels without spheroids (D0, D1: n = 8, D7, D14: n = 7) during cyclic compression-tension up to a maximum strain of 15% in compression (left), and during cyclic shear up to a maximum shear of 30% in torsional shear (right) over 14 days of cultivation.

B: Corresponding average maximum nominal stresses in compression (left), and torsional shear (right) over 14 days of cultivation. Significances were calculated using one-way ANOVA tests if all samples were normally distributed and Kruskal-Wallis tests otherwise, followed by Tukey-Kramer tests for multiple comparisons.

| 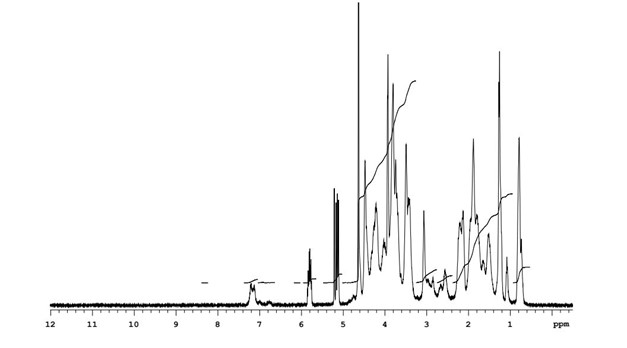 |
| --- |
| ***Figure S9: Proton nuclear magnetic resonance (^1^H-NMR) spectrum of GelAGE.*** |
| ^1^H-NMR spectrum of GelAGE was recorded on a Bruker Biospin 400 MHz spectrometer (Bruker) with deuterium hydroxide (D_2_O) as solvent. |

| 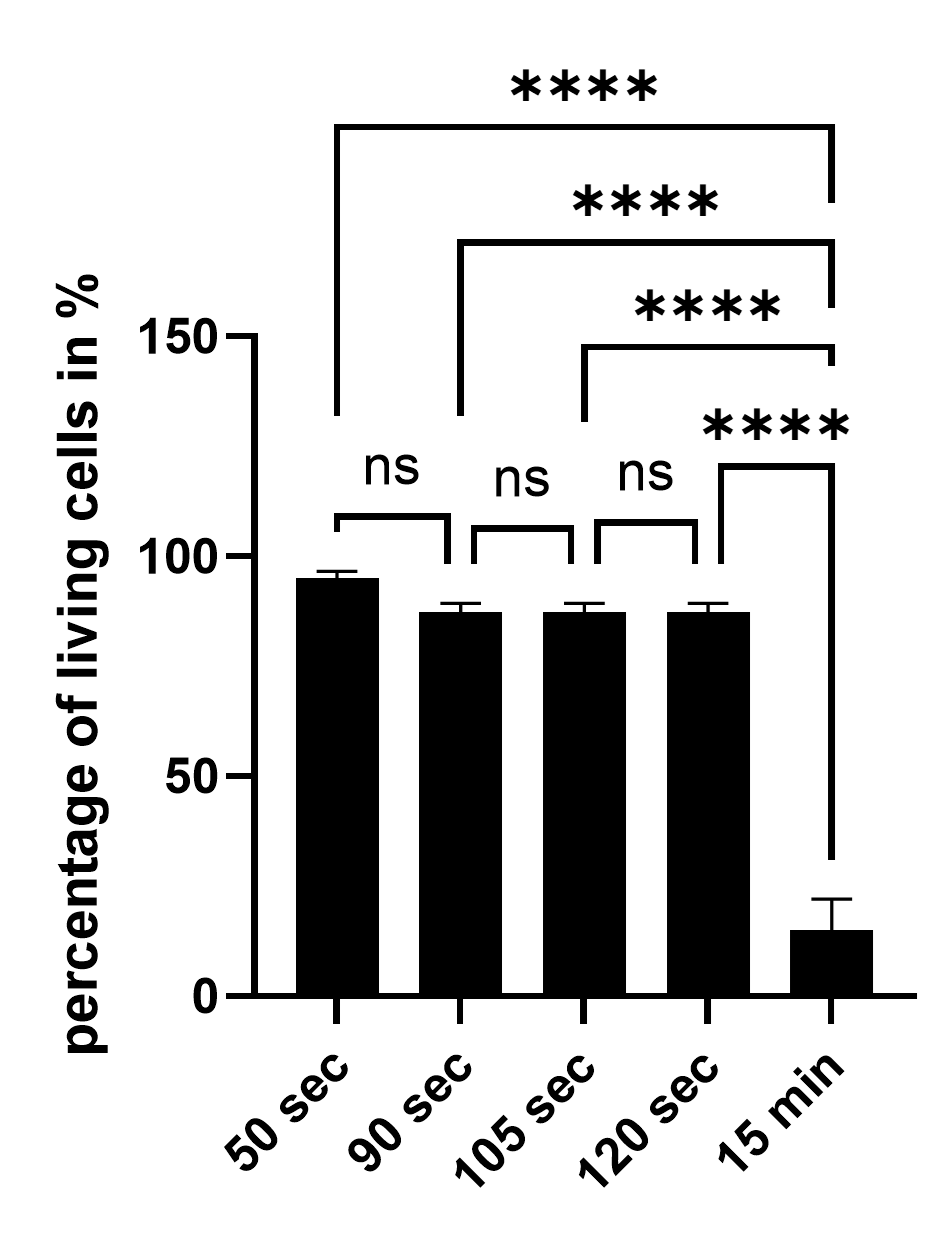 |
| --- |
| ***Figure S10: Survival of glomerular co-culture cells in GelAGE after UV exposure.*** |
| Survival rate of glomerular co-culture consisting of hMC, hGEC, and hPodocytes in GelAGE hydrogels determined with live/dead assay. Imaged 24 hours after UV exposure. Significances were calculated using ordinary one-way ANOVA. Significance value: ****p < 0.0001. |
| GelAGE: allyl-modified gelatin, hMC: human mesangial cells, hGEC: human glomerular endothelial cells, and hPodocytes: immortalized human podocytes, ns: not significant, sec: seconds, min: minutes |

| \| 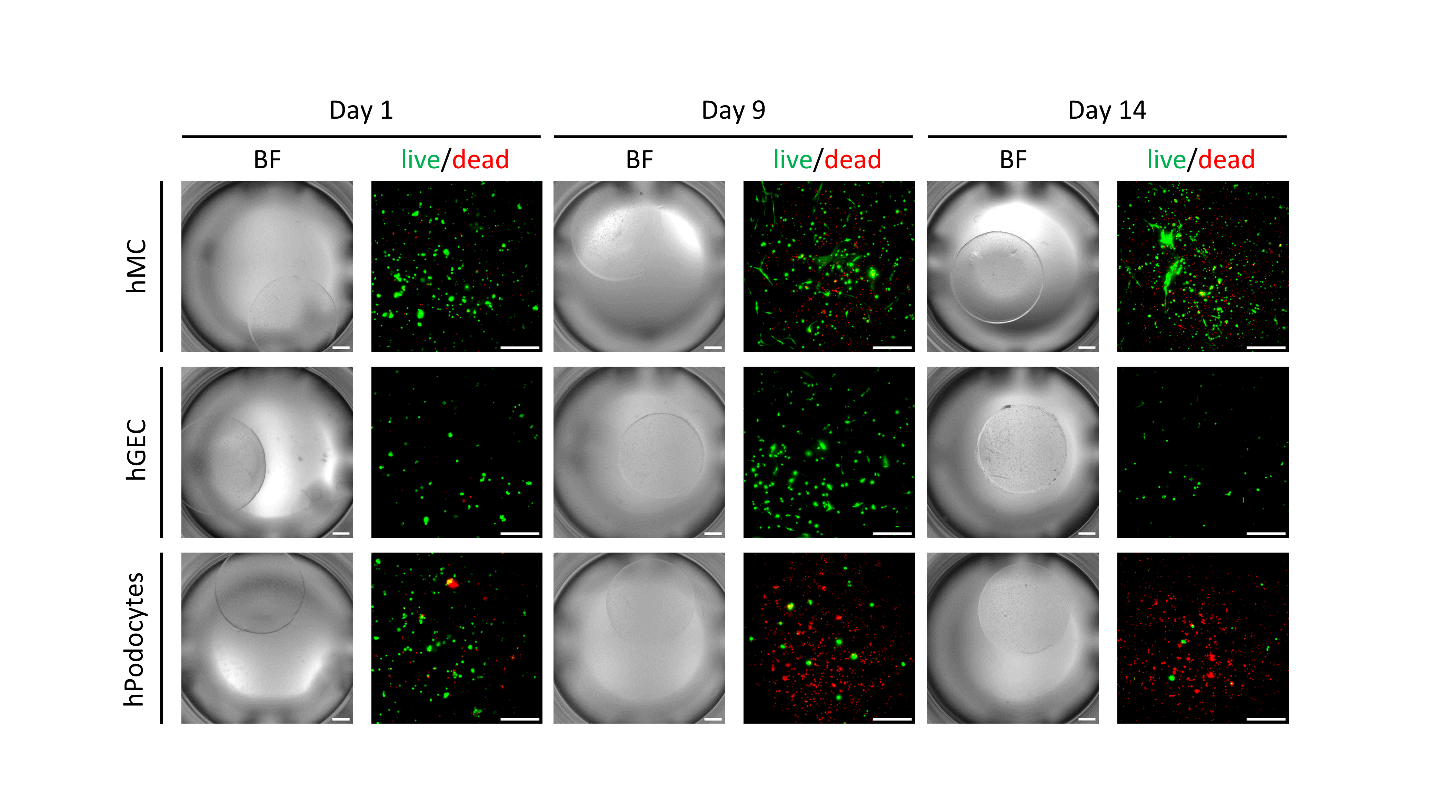 \| \| --- \| \| ***Figure S11: GelAGE as an ECM-mimicking environment for glomerular cells in mono-culture.*** \| \| Bright-field (left panels) and live (green)/dead (red) fluorescence staining (right panels) of hMC, hGEC, and hPodocytes in GelAGE hydrogels. Imaged after one day, nine days, and 14 days of culture. Scale bars: Bright-field: 1,000 µm and live/dead: 300 µm. \| \| ECM: extracellular matrix, hMC: human mesangial cells, hGEC: human glomerular endothelial cells, hPodocytes: immortalized human podocytes, BF: bright-field \| |
| --- | --- | --- | --- | --- |
| 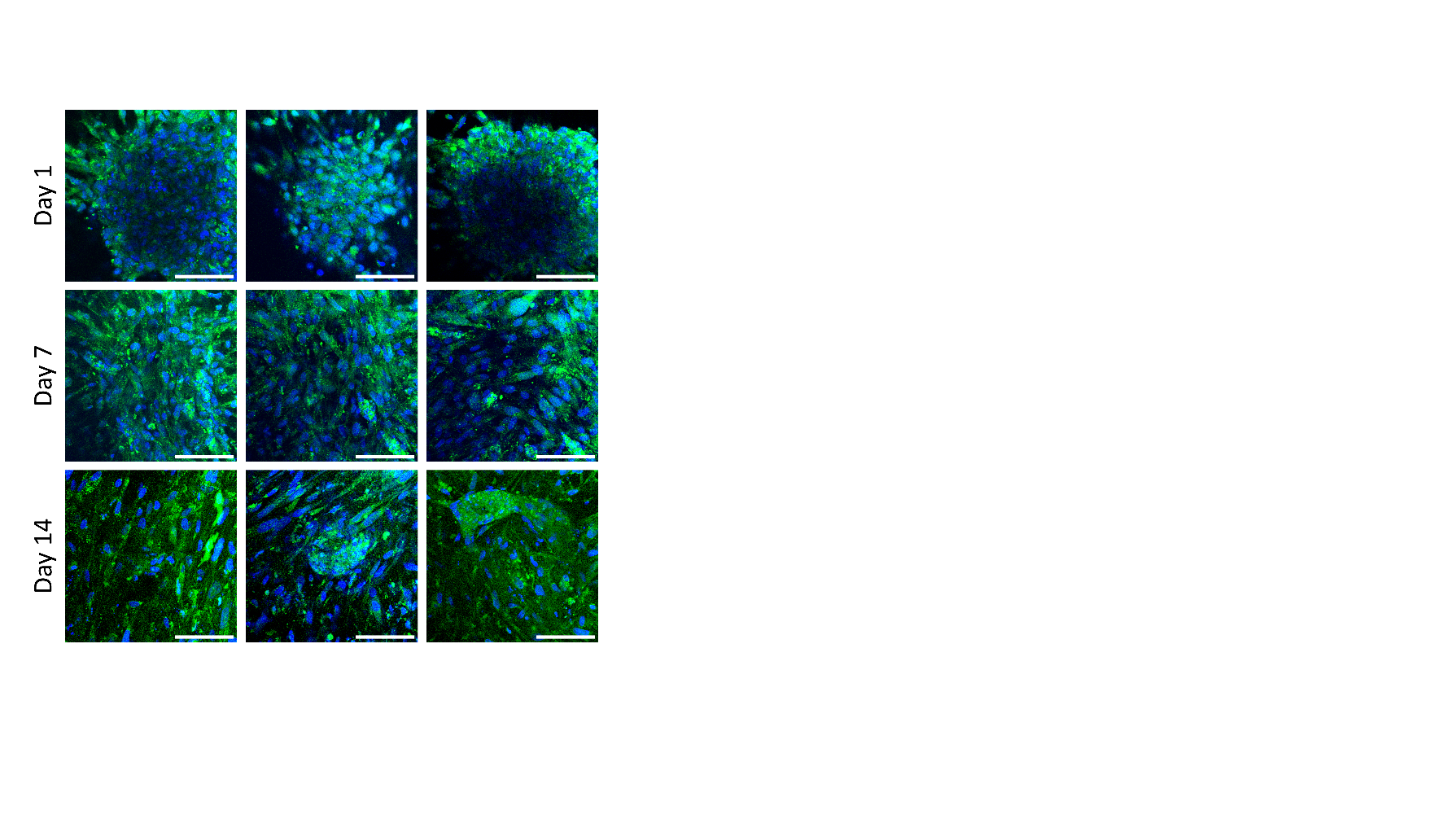 |
| ***Figure S12: Calcein-Hoechst staining of co-culture spheroids in GelAGE***  Calcein-Hoechst staining of co-culture spheroids consisting of hMC, hGEC, and hPodocytes in GelAGE hydrogels. Hoechst (blue) stained all cell nuclei and Calcein (green) stained living cells. Imaged after one day, seven days, and 14 days of culture. Scale bars: 100 µm.  hMC: human mesangial cells, hGEC: human glomerular endothelial cells, hPodocytes: immortalized human podocytes |


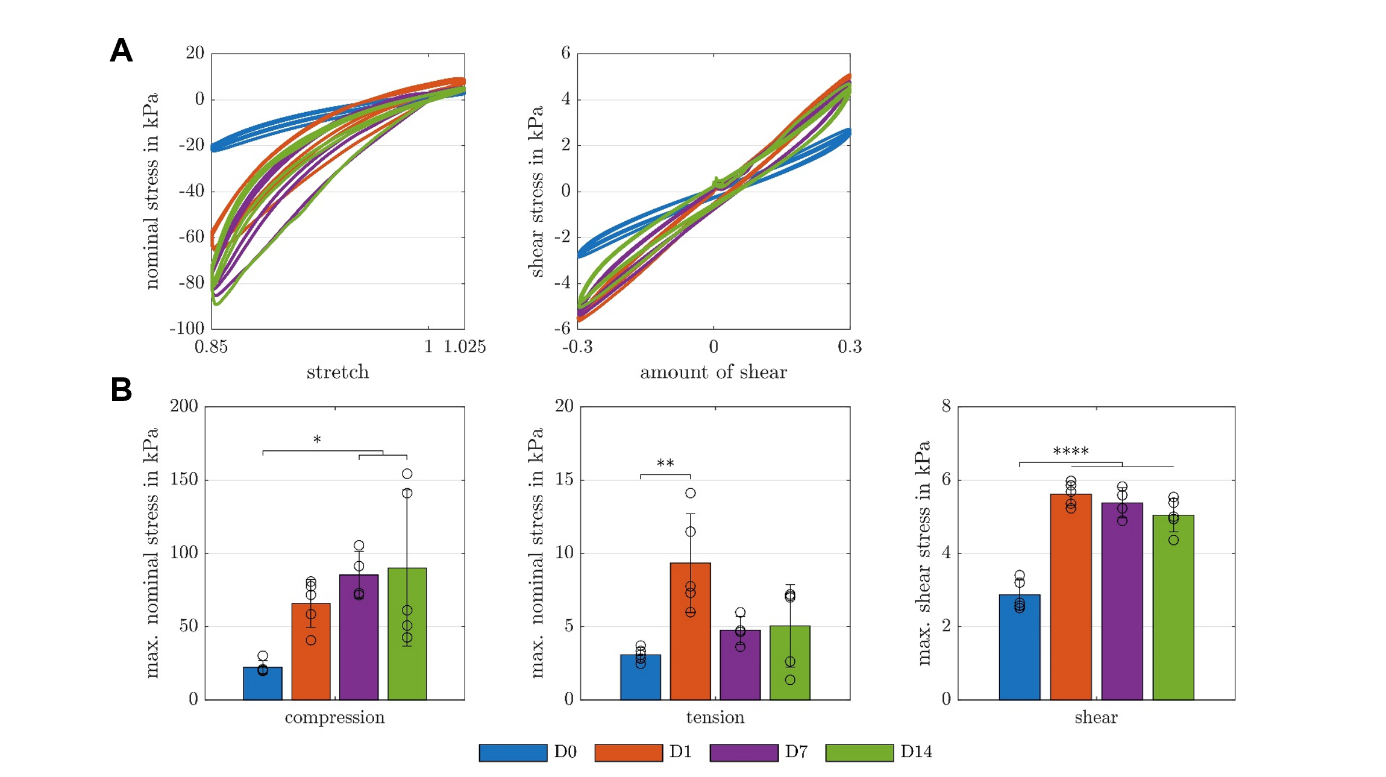


***Figure S13: Mechanical characterization of GelAGE hydrogel without spheroids.***

A: Cyclic loading behavior of GelAGE hydrogels without spheroids (D0, D1, D14: n = 5, D7: n = 4) during cyclic compression-tension up to a maximum strain of 15% in compression, 2.5% in tension (left), and during cyclic shear up to a maximum shear of 30% in torsional shear (center) over 14 days of cultivation.

B: Corresponding average maximum nominal stresses in compression (left), tension (center), and torsional shear (right) over 14 days of cultivation. Significances were calculated using one-way ANOVA tests if all samples were normally distributed and Kruskal-Wallis tests otherwise, followed by Tukey-Kramer tests for multiple comparisons. Significance values: *p < 0.05, **p < 0.01, ****p < 0.0001.
